# Understanding influences on health-seeking behaviours among persons cured of leprosy in the Godokpe community, Volta Region of Ghana: A qualitative descriptive study

**DOI:** 10.1371/journal.pntd.0014753

**Published:** 2026-09-30

**Authors:** Redeemer Joy Duboh, Dickson Kweku Delali, Emmanuel Manu

**Affiliations:** 1 Department of Population and Behavioural Sciences, Fred N. Binka School of Public Health, University of Health and Allied Sciences, Hohoe, Ghana; 2 Department of Theoretical and Applied Biology, Kwame Nkrumah University of Science and Technology, Kumasi, Ghana; 3 Department of Epidemiology and Biostatistics, Fred N. Binka School of Public Health, University of Health and Allied Sciences, Hohoe, Ghana; Marie Adelaide Leprosy Center, PAKISTAN

## Abstract

**Background:**

Leprosy, a curable neglected tropical disease, continues to affect vulnerable populations in low-resource settings despite global control efforts. In Ghana, although leprosy has been eliminated as a public health problem, new cases continue to be detected, and persons cured of leprosy (PCLs) continue to face long-term stigma, disability, and barriers to healthcare in segregated communities. This qualitative descriptive study aimed to understand the knowledge of leprosy, sources of healthcare, and challenges shaping health-seeking behaviours among PCLs in Godokpe, Ho Municipality, Volta Region, Ghana.

**Methodology:**

This qualitative descriptive study explored the knowledge, healthcare sources, and challenges shaping health-seeking behaviours among PCLs. Purposive sampling recruited 20 participants to reach saturation. Semi-structured, face-to-face, in-depth interviews lasting 20–30 minutes were conducted in Godokpe, audio-recorded, transcribed verbatim, and analysed using reflexive thematic analysis in Atlas.ti version 7.5.0, following COREQ guidelines.

**Results:**

This study revealed four central themes. Knowledge of Leprosy showed predominant supernatural attributions, including bewitchment, ancestral curses, and contaminated food, alongside rare bacterial recognition, with signs such as skin lesions, ulcers, nodules, limb deformities, loss of sensation, and body pain. Social Treatment and Stigma reflected two distinct patterns: ongoing, unevenly resolved interpersonal avoidance by family and community, contrasted with a documented shift toward equal treatment of PCLs at health facilities over time. Health-Seeking Behaviour reflected a pluralistic pattern, drawing on biomedical facilities such as the Ho Polyclinic and Ho Teaching Hospital, herbal remedies, self-medication, and spiritual or church-based healing based on perceived severity. Challenges Influencing Health-Seeking encompassed logistical barriers such as distance, transport costs, drug unavailability, and informal charging for nominally free medication; physical limitations such as mobility impairment, painful or wounded legs, and visual loss; and socioeconomic constraints such as unemployment, financial hardship, low allowances, donor dependence, and the prioritisation of food over healthcare.

**Conclusion:**

This study revealed persistent supernatural attributions of leprosy, an uneven trajectory of stigma reduction, pluralistic health-seeking, and layered barriers, including a documented gap between free-care entitlements and informal charging in practice, among PCLs in Godokpe. Addressing these gaps requires verification of fee-exemption entitlements at the facility level alongside sustained family- and community-level engagement, advancing Sustainable Development Goal (SDG) 1 on poverty reduction, SDG 3 on good health and well-being, and SDG 10 on reduced inequalities.

## Author summary

Leprosy remains an important public health concern in many low-resource settings, even after successful treatment. In this study, we explored how persons cured of leprosy living in Godokpe, a settlement in Ghana’s Volta Region, seek healthcare and the challenges and support they experience after cure. Through in-depth interviews, we found that many participants still associated leprosy with spiritual causes such as curses and bewitchment, while only one participant understood that it is caused by bacteria. Participants used different forms of healthcare, including hospitals, herbal medicine, self-medication, and church-based healing, depending on their beliefs, finances, and health conditions. We also found that how persons cured of leprosy are treated by others has changed over time in important ways. While some participants still experience being avoided by family or community members, several independently described being treated equally to other patients at health facilities today, a marked change from being seated separately or treated last in the past. At the same time, poverty, unemployment, disability, transportation difficulties, and drug shortages, including being unexpectedly charged for medication that was expected to be free, continued to strongly influence healthcare access. Some participants struggled to walk to health facilities because of wounds, pain, or visual impairment, while others prioritised food over healthcare because of financial hardship. Our findings show that the effects of leprosy continue long after medical treatment is completed, alongside real progress in how affected persons are treated within the health system. We believe this study highlights the need for stronger post-cure support, continued family- and community-level stigma reduction, verification of free-care entitlements, disability care, and social protection programmes to improve the wellbeing and inclusion of persons cured of leprosy.

## Introduction

Leprosy, a chronic infectious disease caused by *Mycobacterium leprae*, continues to affect vulnerable populations in low- and middle-income countries despite being fully curable with multidrug therapy (MDT) [1]. The infection targets the skin, peripheral nerves, upper respiratory tract mucosa, and eyes, often leading to irreversible disabilities when diagnosis is delayed [1]. In 2024, global new cases reached 172,717, reflecting a 5.5% decline from 182,815 in 2023, yet 9,397 involved children and 9,157 presented with grade-2 disabilities (G2D), clear indicators of persistent transmission and late detection [2]. Women accounted for 69,394 cases (40.2%), while Brazil, India, and Indonesia together reported the majority, with over 120 countries still endemic [2]. The WHO’s Global Leprosy Strategy 2021–2030 seeks zero leprosy through intensified surveillance, preventive chemotherapy like single-dose rifampicin for contacts, and aggressive stigma reduction, targeting a 70% reduction in new cases and 90% fewer G2D cases by 2030 [3]. Persistent challenges, including poverty, cultural misconceptions portraying leprosy as a curse or incurable affliction, climate change, and migration, sustain transmission and complicate control efforts [4].

Africa accounts for about 40% of the global burden of Neglected Tropical Diseases (NTDs), with about 600 million individuals requiring treatment [5]. The continent recorded 19,171 new cases in 2024, with poverty accounting for diagnostic delays resulting in elevated disability rates [2,6]. Programs such as Post-Exposure Prophylaxis for Leprosy (PEP4LEP) have screened over 36,531 contacts since 2018 in Ethiopia, Tanzania, and Mozambique, detecting skin conditions in roughly half and advancing community-based prevention [7]. According to World Health Organization data, Ghana recorded 278 new leprosy cases (8.075 per 1,000,000 population) in 2024, with a total of 373 cases (prevalence of 10.834 per 1,000,000 population) remaining on treatment registers as at the end of the year [2]. Of the new cases, 42 (15.1%) presented with G2D, 8 (0.649 per 1,000,000 child population) were among children under 15 years, and 126 (45.3%) were female [2]. The elimination goal for leprosy in Ghana is a prevalence of <1 new case with G2D per 10,000 population [8].

The Volta Region illustrates these national dynamics, with incidence climbing from 9.48 per million in 2019 to 16.50 in 2021 before dropping to 2.34 in 2023 [9]. Between 2019 and 2023, 82 new cases were documented, predominantly multibacillary (80 of 82) and among older adults, with Hohoe Municipality reporting the highest tally at 17 [9]. This concentration of historical caseload in the Ho area has left a lasting social legacy: the historic Ho Leprosarium continues to serve as a treatment hub, while nearby segregated communities like Godokpe house cured persons who face enduring social exclusion, often spending decades there post-cure due to family rejection, community discrimination, and internalised shame that limit reintegration into broader society [10]. In-depth accounts from residents reveal how stigma manifests in restricted movements, denied employment opportunities, and fractured relationships, compelling many to rely on the village’s limited resources for survival despite being non-infectious [10,11]. These patterns underscore how cultural beliefs viewing leprosy as a spiritual curse or hereditary affliction further deter health-seeking, with affected individuals often turning first to traditional healers before biomedical options, exacerbating delays and complications even among the cured [11,12].

Existing literature exposes critical shortcomings in post-cure support for persons cured of leprosy (PCLs), particularly where stigma persists after cure and discourages engagement with mainstream health services [9,11]. In African contexts like PEP4LEP sites, low awareness and gender barriers compound issues, while in Ghana, PCLs in isolated villages endure economic marginalisation, self-stigma, and beliefs framing leprosy as hereditary or spiritual, all limiting access to general care [11]. While studies in the Ho area have examined stigma and social exclusion among residents of Godokpe [10] and barriers to contact tracing for early detection [11], these focus primarily on active disease dynamics, general affected populations, or pre-cure/prevention phases. They provide valuable context on persistent stigma, cultural beliefs, and community isolation but offer limited depth into how cured individuals, rendered non-infectious after the start of MDT, navigate ongoing influences such as self-stigma, disability management, economic constraints, and fear of discrimination when deciding to access healthcare for secondary conditions, mental health support, or routine needs. This gap hinders the development of tailored interventions that address the unique vulnerabilities PCLs face long after treatment completion, including reduced quality of life, social reintegration challenges, and underutilization of mainstream health services.

This study addresses these gaps by exploring the influences on health-seeking behaviours among persons cured of leprosy in Godokpe, Volta Region of Ghana. Specifically, the study aims to assess their knowledge of leprosy, identify the healthcare sources they use, and examine the challenges influencing their health-seeking behaviours.

## Methods

### Ethics statement

Ethical approval for this study was obtained from the University of Health and Allied Sciences Ethics Review Committee, with Protocol Identification Number UHAS-REC A.9 [1] 20–21, which constituted the substantive ethical oversight for the study. In addition, as a matter of community protocol and access rather than ethical oversight, courtesy permission was sought from the Foundation, the community-based administrative body that has managed residency and welfare matters in Godokpe since the settlement’s establishment. The Foundation is a formally recognised non-governmental organisation; however, its registration is administrative and welfare-oriented rather than an ethics-review mandate, and it was not treated as holding statutory or ethics-review authority over participants. UHAS-REC approval was the binding ethical authorisation for this study. The study adhered to the ethical principles outlined in the Declaration of Helsinki [13] and the guidelines of Good Clinical Practice (GCP). Written informed consent was obtained individually from each participant before enrolment, and participation was entirely voluntary; individuals who declined or withdrew consent at any stage were excluded without penalty. Given the small and identifiable nature of the Godokpe community, care was taken to reduce the risk of deductive disclosure: participant quotations are attributed only to a study identification number, sex, and age (or age category), and no other potentially identifying details (such as specific family circumstances or exact duration of residence) are reported alongside quotations.

### Study design

This study adopted a qualitative descriptive design to explore the knowledge, healthcare sources, and challenges shaping health-seeking behaviours among persons cured of leprosy (PCLs). Qualitative description is well-suited to producing a comprehensive, data-near summary of a phenomenon in participants’ own words and categories, without requiring the interpretive or philosophical commitments of approaches such as phenomenology or grounded theory [14]. Data were analysed using reflexive thematic analysis [15]. Reporting adhered to the Consolidated Criteria for Reporting Qualitative Research (COREQ) to ensure transparency in context, methods, analysis, and findings [16].

### Study setting

Godokpe is located in Ho, the capital of the Volta Region of Ghana, which is bordered by Adaklu-Anyigbe District to the south, Hohoe Municipality to the north, South-Dayi District to the west, and the Republic of Togo to the east. According to Sottie and Darkey [10], the settlement traces its origins to the Ho leprosarium, established in 1926, where patients historically remained admitted for extended periods and were frequently abandoned by their families and unable to reintegrate into their communities after cure. Following the introduction of Multi-Drug Therapy (MDT), which shortened treatment duration, the leprosarium was converted into a polyclinic and its residential wards closed, leaving many cured persons, particularly those rejected by their families, without accommodation. These individuals settled near the polyclinic, in an area (initially known as Freetown) that expanded over time through intermarriage and the arrival of others who could not return to their home communities. When land disputes later threatened the settlement, a philanthropist secured funding from a German non-governmental organisation to purchase the land and construct houses for residents, formally establishing what is now known as Godokpe. This history of institutional origin, prolonged segregation, and external, rather than governmental, provision of housing is relevant to understanding both the setting’s suitability for this study and the community-level approval process described under Ethics Considerations. Interviews were conducted in person within Godokpe, in private spaces arranged with participants to protect confidentiality.

### Study population

The target population comprised persons cured of leprosy residing in Godokpe, Ho Municipality, Volta Region, Ghana. All participants had completed multi-drug therapy (MDT) and had been long-term residents of Godokpe for at least 18 years, rather than recently treated individuals; this population was therefore expected to reflect the health-seeking knowledge, sources, and challenges shaped by years of post-cure life in the settlement, which may differ from those of persons more recently treated for leprosy.

### Inclusion and exclusion criteria

Eligible participants were PCLs aged ≥18 years, residing in Godokpe, and available during the data collection period. Exclusion criteria included severe or critical health conditions preventing effective participation and temporary absence from the community during the study period.

### Sampling and sample size

Purposive sampling was used to recruit participants with relevant experiences and knowledge, consistent with qualitative priorities for depth rather than representativeness [17]. This approach may be particularly valuable when the population is small and clearly meets the study’s requirements, or when refining samples from other methods, as it relies on the researcher’s expertise to select the most relevant participants [18]. As detailed in the eligibility criteria section, participants were selected based on their status as long-term cured residents of Godokpe. Eligible PCLs were identified through a resident list held by the Cured Lepers Foundation, in collaboration with community leaders, who helped confirm residents’ continued presence in Godokpe and their willingness to be approached. In total, twenty persons cured of leprosy were recruited, consistent with recommendations of 10–20 participants for studies seeking to explore and understand lived experience [19]. This sample size was further supported by the concept of information power, whereby the more relevant information a sample holds, the fewer participants are required; a sample of this size was considered appropriate given the study’s narrowly defined aim, the specificity of a single, well-defined resident population, and the depth of dialogue achieved in interviews [20]. Recruitment continued until thematic saturation was reached, with saturation considered achieved when successive interviews yielded no new themes.

### Pilot interviews

Four pilot in-depth interviews (two males, two females) assessed interviewer competence and interview guide clarity, appropriateness, and cultural sensitivity. Feedback prompted minor rephrasing for improved comprehension; pilot participants were excluded from the main sample.

### Data collection instrument and procedure

Data were collected using a semi-structured interview guide developed based on the study’s objectives, covering four broad domains, each probed with follow-up questions to elicit further detail. The first addressed participants’ knowledge and perceived causes of leprosy. The second explored participants’ experiences of social treatment and stigma, including how they were treated by family, by the wider society, and by staff when accessing care. The third addressed current health-seeking behaviour, sources of care, and the barriers encountered in accessing public institutions such as hospitals, schools, and banks. The fourth, distinct from the third, asked participants to recall their experience around the time of their original leprosy diagnosis; this retrospective material is presented separately from participants’ accounts of current health-seeking practices. The guide closed with broader reflective questions on participants’ overall experience of living in the community and their views on how persons affected by leprosy should be treated in Ghana. The interviews were conducted face-to-face by two trained research assistants with individual participants. The interview guide was originally developed in English and was read to participants during the interviews. For participants who could not understand English, the questions were translated into the local dialect (Ewe) to facilitate comprehension. With the consent of the participants, all in-depth interviews (IDIs) were audio-recorded. The IDIs were used to explore participants’ experiences and perspectives regarding factors influencing their health-seeking behaviour in daily life, while also providing them with the opportunity to speak privately and openly during the interviews. In addition, the open-ended nature of the questions allowed participants to express their views freely, enabled the researchers to probe responses for clarification, and facilitated deeper discussion of emerging issues. Each interview lasted approximately 20–30 minutes; this duration was considered appropriate given the study’s descriptive rather than exhaustive-exploratory aim, and was also intended to minimise fatigue among participants, many of whom were older adults with mobility or health-related limitations.

### Data analysis

Following data collection, the audio-recorded in-depth interviews were transcribed verbatim to ensure that participants’ responses were accurately captured. Interviews conducted in the local dialect (Ewe) were first transcribed and subsequently translated into English to facilitate analysis. The transcripts were reviewed alongside the audio recordings to verify accuracy and completeness. All transcripts were then imported into Atlas.ti Version 7.5.0 to support the systematic organisation and analysis of the qualitative data. The analysis followed the phases of reflexive thematic analysis [15], applied iteratively rather than as a strict linear sequence. Initially, the researchers familiarised themselves with the data by repeatedly reading the transcripts to gain a comprehensive understanding of participants’ accounts and perspectives regarding health-seeking behaviour. During this stage, preliminary ideas and observations were documented.

Subsequently, open coding was conducted within Atlas.ti, where meaningful segments of text were identified and assigned descriptive codes that reflected key ideas expressed by participants. The coding process was iterative, with codes refined as new insights emerged. To enhance the rigour and credibility of the analysis, a second researcher independently reviewed a subset of the transcripts and the initial coding scheme. Any discrepancies in coding were discussed and resolved through consensus, leading to the development of a consistent coding framework applied across all transcripts. Following initial coding, similar codes were grouped to form broader categories based on recurring patterns within the data. These categories were further examined and refined to develop themes that captured the underlying meanings and shared experiences of the participants. The themes were then reviewed and compared across transcripts to ensure consistency and to confirm that they accurately represented participants’ accounts.

Throughout the process, Atlas.ti facilitated efficient data management, retrieval of coded segments, and organisation of codes into thematic networks. The final themes were interpreted in relation to the study objectives and used to present the findings on the influences of health-seeking behaviours among persons cured of leprosy. During revision, all quotations presented in the Results section were audited against the original interview transcripts. This audit identified and corrected instances where quotations had combined participants’ answers to different interview questions without disclosure, along with a small number of attribution and transcription inaccuracies. Corrected quotations reflect participants’ verbatim responses to the specific question each quotation illustrates.

### Trustworthiness of the study

The trustworthiness of this study was ensured by addressing the criteria of credibility, dependability, confirmability, and reflexivity which are widely recognised as essential components of rigour in qualitative research.

**Credibility:** It was enhanced through prolonged engagement with the data and repeated reading of transcripts to ensure a deep understanding of participants’ accounts and perspectives. The use of in-depth interviews allowed participants to provide rich and detailed accounts of their experiences, thereby strengthening the authenticity of the data. Audio recording and verbatim transcription further ensured that participants’ responses were accurately captured. In addition, selected transcripts and coding processes were reviewed by all the authors, and discrepancies were discussed and resolved to enhance the credibility of the findings.

**Dependability:** It was ensured by maintaining a clear, systematic, and well-documented research process. Detailed descriptions of the data collection and analysis procedures were provided, including the use of a semi-structured interview guide and reflexive thematic analysis. The coding process was iterative, and a consistent coding framework was applied across all transcripts. Peer review of portions of the data and coding scheme further strengthened the dependability of the study.

**Confirmability:** It was achieved by ensuring that all interpretations were grounded in the data. An audit trail was maintained throughout the research process, including transcripts, coding decisions, and theme development. In addition, the use of direct quotations in the presentation of findings provided evidence that the results were derived from participants’ accounts.

**Reflexivity:** The two research assistants who conducted the interviews were one male and one female, both trained before data collection and affiliated with academic institutions in Ghana (University of Health and Allied Sciences and Kwame Nkrumah University of Science and Technology), with backgrounds in public health research rather than clinical care provision at any facility accessed by participants. Both were native Ewe speakers, which allowed interviews to be conducted directly in participants’ first language without reliance on a third-party interpreter, reducing a potential source of translation-related distortion during data collection itself. As Ghanaian nationals, the research assistants were broadly familiar, through their own cultural context, with the explanatory frameworks, such as bewitchment and ancestral curses, that featured prominently in participants’ accounts of leprosy’s causes, without holding these beliefs themselves as explanations for leprosy specifically. This shared cultural familiarity may have supported rapport and reduced participants’ need to over-explain culturally embedded concepts, while the researchers’ academic and biomedical disciplinary orientation may still have shaped which aspects of participants’ accounts were probed further and how these explanations were interpreted during coding. To manage this, the research team held debriefing discussions after each interview and recorded written memos capturing their own initial reactions and assumptions before formal coding began; these memos and debriefing notes were revisited during team coding-review meetings, where individual assumptions about the plausibility or meaning of participants’ explanatory frameworks were explicitly raised and discussed, rather than allowed to pass unexamined into the coding scheme.

## Results

### Socio-demographic characteristics of persons cured of leprosy

Table 1 presents background characteristics of the persons cured of leprosy (PCLs) who participated in the study. Amongst the 20 participants, 15 were females, and 5 were males. Thirteen were aged 60 years and above, and five could not recall their exact age; no estimated range is reported for these participants, as neither the participants nor available records provided a reliable basis for estimation, and the research team judged that an approximation risked overstating precision that did not exist. Half of the participants were widowed: nine were widows, and one was a widower. All participants who took part in the study were of Ewe ethnicity. Nineteen of the participants were unemployed, and one was engaged in farming. Out of the 20 participants, nineteen were Christians, and one was Muslim.

**Table 1:** Socio-demographic characteristics of persons cured of leprosy.

Variables	Frequencies (20)
**Age**	
20-29	0
30-39	1
40-49	0
50-59	1
60+	13
Unknown	5
**Sex**	
Male	5
Female	15
**Marital Status**	
Unmarried	4
Married	4
Divorced	2
Widow	9
Widower	1
**Ethnicity**	
Ewe	20
**Occupation**	
Unemployed	19
Farmer	1
**Religion**	
Christian	19
Muslim	1

### Thematic findings

Table 2 presents three main themes across participant groups, with sub-themes derived from iterative coding.

**Table 2:** Thematic findings.

Major Theme	Sub-Theme	Codes
**Knowledge**	General Causes	Superstition
		Bacterial Infection
	Signs and Symptoms	Loss of Stimuli
		Ulcer
		Lesions
		Scars
		Thickening of limbs
		Stiffening of limbs
		Body pain
		Skin nodules
**Social Treatment and Stigma**	Historical Segregation and Fear-Driven Avoidance	Isolation at symptom onset
		Fear-driven avoidance by others
		Family distancing
		Residual social avoidance
	Institutional Shift Toward Equal Treatment	Segregated seating (past)
		Differential/priority treatment (past)
		Equal treatment (present)
**Health Seeking Behaviour**	Health Seeking Sources	Health facility
		Herbal medicine
		Self-medication
		Church
		Orthodox
**Challenges**	Structural Barriers to Health-Seeking	Distance to health facilities
		Unavailability of drugs
	Physical Impairment	Visual impairment
		Painful and wounded legs
	Socio-economic Challenges (Direct and Indirect Costs)	Financial Challenges
		Unemployment

### General knowledge of leprosy

Under the theme Knowledge of Leprosy, two key subthemes emerged from the analysis: General Causes and Signs and Symptoms. These subthemes reflect participants’ explanations of what they believed caused leprosy and how they recognised the disease through its physical manifestations. The General Causes subtheme captures participants’ perceptions regarding the origin of leprosy. Some participants attributed the disease to bacterial infection, demonstrating awareness of the biomedical explanation of leprosy. However, other participants associated the disease with superstition or spiritual influences, reflecting the persistence of socio-cultural beliefs in the interpretation of illness. These differing explanations highlight the coexistence of biomedical knowledge and traditional beliefs in shaping participants’ understanding of the disease. The following quotes summarise their responses,

*“Some people were bewitched with it, and with others, their forefathers killed [certain living] things they were not supposed to kill during war, and it intends to infect current and future generations with leprosy”* [Participant 1, female, 86 years old].

*“Leprosy is in such a way that everyone has their unique way of getting infected, but I do not believe that one can just be infected with it unless he or she has been bewitched by someone”* [Participant 11, female, 85 years old].

Another participant explained how her entire family got infected after eating a yam brought home by her grandfather. She narrated:

*“From my experience, my grandfather, who was a farmer, came home with yams, and after eating them, we all got infected with leprosy”* [Participant 9, female, 90 years old].

This account was coded under General Causes alongside explicitly supernatural attributions because, although it names a specific food, the underlying logic is not one of biomedical contamination (for example, aflatoxin or infection) but of a mystical, family-wide transmission triggered by an act of eating; the yam functions here as a vehicle for a curse-like mechanism rather than as a biomedical vector, consistent with the participant’s broader framing of the illness as something that happened “to the family” rather than to an individual through exposure.

Only one participant linked bacteria as being the cause of leprosy. The participant vividly explained the strain of bacteria that infects humans with leprosy. He recounted:

*“There is a bacterium called Mycobacterium leprae that infects an individual with leprosy. The symptoms show after 2-3 years. It [the bacteria] is known to be an airborne disease, affecting vessels and muscles, leading to deformities and ulcers. It [the bacteria] also makes you lose your sense of touch due to damage to sensory neurons. Your hands can also have growths with fluid discharges”* [Participant 3, male, 72 years old].

Participant 3’s account is notable for its level of biomedical specificity and stood apart from all other accounts in this study, which drew almost exclusively on supernatural or lay explanatory frameworks. This is plausibly explained by his occupational history: he reported having worked as an orderly at the leprosarium, a role that would have given him sustained, direct exposure to clinical staff and medical terminology unavailable to most other participants. This suggests that biomedical understanding of leprosy in this community may be unevenly distributed according to occupational and institutional proximity, rather than uniformly absent, and that differential exposure to health education or provider contact, rather than a wholesale absence of biomedical knowledge in the community, may better explain the pattern observed here.

The Signs and Symptoms subtheme represents participants’ descriptions of the physical changes they experienced or observed as indicators of the disease. Participants reported several symptoms, including loss of sensation, skin lesions, ulcers, scars, and skin nodules, which they recognised as early or visible manifestations of leprosy. Some also described more advanced physical complications such as thickening of limbs, stiffening of limbs, and body pain, which often affected their mobility and daily functioning. The comments below summarise participants’ knowledge of the signs and symptoms of leprosy,

*“The disease is not infectious; you will just start seeing lesions that will make your skin different from what you previously had”* [Participant 2, female, 75 years old].

*“...It [leprosy] leads to deformities and ulcers. It also makes you lose your sense of touch due to damage to sensory neurons. Your hands can also have growths with fluid discharges”* [Participant 3, male, 72 years old].

*“It [leprosy] starts with thickening and stiffening of your limbs and skin, leading to loss of stimuli or sense of touch”* [Participant 12, female, 79 years old].

### Social treatment and stigma

Under the theme Social Treatment and Stigma, two subthemes emerged: Historical Segregation and Fear-Driven Avoidance and Institutional Shift Toward Equal Treatment. These subthemes distinguish between stigma experienced at the interpersonal and family level and a documented pattern of institutional practice that participants described as having changed over time.

The Historical Segregation and Fear-Driven Avoidance subtheme captures participants’ accounts of being isolated or avoided by others, most often at the onset of their illness, out of fear of infection. Some participants described this as an early and later-resolved experience, while others described it as an avoidance that had continued into the present. The following quotes summarise their responses,

*“I got isolated from the onset of the infection, and was not allowed to go closer to anybody”* [Participant 3, male, 72 years old].

*“Initially, they never got closer because they feared being infected as well”* [Participant 13, female, age unknown].

*“They stigmatize me by not getting closer to me due to my current state”* [Participant 15, female, 80 years old].

One participant described this avoidance specifically within her family, while noting that the same distancing did not extend to how she was received in health settings. She narrated:

*“Some no longer get closer or relate to you when they notice you have this disease, but when you come to the clinic or hospital, they receive you gladly”* [Participant 1, female, 86 years old].

The Institutional Shift Toward Equal Treatment subtheme reflects a distinct and more specific pattern: several participants, independently of one another, described a documented change in how PCLs were formally treated at health facilities, moving from segregated or prioritised treatment in the past to equal treatment alongside non-affected patients in the present. The following quotes illustrate this shift,

*“We were made to sit at separate places away from people not infected with leprosy, but now we sit together with uninfected people for medical care and consultation”* [Participant 10, male, 75 years old].

*“They attend to all of us equally, as we have come to seek treatment, but formerly, they used to treat us first before those who have not been affected by leprosy”* [Participant 14, male, 63 years old].

*“In the beginning, they used to attend to us first before attending to those who have never been affected by leprosy; now we are being treated equally”* [Participant 20, male, 80 years old].

That three participants independently described the same institutional practice and its subsequent change suggests this reflects an actual shift in facility-level policy or norms over time, rather than an isolated perception, and may be worth situating against the broader national and WHO-driven leprosy destigmatisation efforts described in the Introduction.

### Health-seeking behaviour

Under this theme, the subtheme health-seeking sources emerged from participants’ narratives. Unless otherwise indicated, the accounts presented here describe participants’ current, ongoing health-seeking practices as PCLs, that is, how they now seek care for general health needs, secondary conditions, and leprosy-related complications, rather than the historical process through which they were originally diagnosed with and treated for leprosy. This subtheme highlights the different avenues through which persons cured of leprosy sought treatment or relief when they experienced symptoms or health-related concerns. The findings in this study revealed that participants relied on a variety of healthcare options, reflecting a pluralistic approach to health-seeking. Several participants reported seeking care from health facilities, particularly formal government-run medical institutions (referred to by some participants as “orthodox” healthcare, meaning conventional biomedical facilities such as the Ho Polyclinic and Ho Teaching Hospital, as distinct from herbal or spiritual sources) where they could receive professional diagnosis and treatment. These orthodox healthcare services were often considered reliable because they involved trained health professionals and recognised medical treatments for leprosy and other illnesses. However, participants did not rely solely on biomedical care. Some reported the use of herbal medicine, obtained either from traditional healers or prepared locally, which they believed could help manage symptoms or promote healing. In addition, self-medication emerged as another practice among some participants, where individuals used over-the-counter drugs or home remedies without consulting a healthcare professional. Participants also mentioned seeking spiritual support through the church, including prayer and faith-based healing practices, particularly when illness was perceived to have spiritual or supernatural influences. Participants’ reasons for moving between these sources varied and did not reduce to a single logic. Some described severity-based escalation within the biomedical system itself, moving from the local clinic to larger facilities such as the Ho Polyclinic or Ho Teaching Hospital as their condition worsened. Others moved away from biomedical care for reasons unrelated to symptom severity, including a loss of trust following an experience of being denied medication, or a stated personal preference for church-based prayer over hospital care despite no reported barrier to access. Rather than a single severity-based hierarchy governing all participants’ choices, this suggests that health-seeking in this community is shaped by several distinct and sometimes co-occurring logics: escalation by severity within biomedical care, disengagement following negative experiences with that same system, and individual preference independent of either.

Some participants saw it as a necessity to visit hospitals and clinics for healthcare. For this category of respondents, they had no alternative to healthcare aside from visiting government hospitals and clinics. The following quotes summarise their responses,

*“I do seek health care from the Ho Polyclinic. And I do go to the hospital only when I am sick, but have not been to the hospital for the past two years”* [Participant 6, female, unknown age].

*“I do go to the clinic meant for the leprosarium, [but] when my [health] condition worsens, I either go to the Ho Polyclinic or the Ho Teaching Hospital”* [Participant 12, female, 79 years].

Some participants also described using traditional herbal medicine as a complement to, rather than a substitute for, biomedical care. As illustrated below, herbal medicine was typically used alongside continued access to the hospital for more serious cases, with participants turning to herbal remedies specifically for milder situations.

*“I do rely on herbal medicines as well for my health”* [Participant 8, female, unknown age].

Self-medication was reported by several participants as a practice used when symptoms were considered mild or when participants had lost confidence in the biomedical system, whether because of unavailable medicines, unexpected charges, or perceived unresponsiveness. In these instances, participants described buying medication from either pharmacies or licensed chemical shops without a prescription rather than returning to the hospital or clinic. One participant explained why he self-medicates:

*“I have decided not to go to the hospital again because I went and was not given any medication. Any time I feel unwell, I would rather visit a pharmacy. We are not to make any payment whenever we visit the hospital, but this time, anytime we visit, they demand money from us for medications”* [Participant 19, male, 73 years old].

With religion being a central part of most Ghanaians’ lives, some participants were of the view that prayers could help cure them of leprosy. One female participant (of the 20 in this study), who described the church as her primary source of health-seeking, explained,

*“I do not have any place to go aside from church... I do not like going to the hospital, although I do not have anything that prevents me from accessing the hospital”* [Participant 15, female, 80 years old].

### Health-seeking challenges faced by participants

Under the theme challenges influencing health-seeking behaviours, three key subthemes emerged from the analysis: structural barriers, physical impairment, and socio-economic challenges. These subthemes reflect the different barriers participants encountered in their efforts to access healthcare services and maintain their well-being.

Structural Barriers to Health-Seeking capture difficulties directly related to accessing healthcare services, spanning two distinct but interacting layers. The first is an access barrier: the physical distance to health facilities, compounded by the cost and availability of transport, made it difficult for participants to seek timely medical attention. The second is a service gap: even when participants reached a facility, the unavailability of drugs meant they sometimes had to return home without receiving the medication they required, and in some cases, medication was withheld unless payment was made, despite this contradicting the free-care policy participants expected. Beyond the immediate inconvenience, this service gap appeared to have a behavioural impact, as repeated experiences of travelling to a facility only to leave empty-handed, or being asked to pay unexpectedly, eroded participants’ trust in the reliability of care and reduced their motivation to attempt the journey again, a pattern also evident in participants’ descriptions of turning to self-medication once biomedical care had disappointed them. The following quotes summarise their responses,

*“Walking to the hospital has been my challenge, and I am only able to access the hospital through commercial means of transport”* [Participant 3, male, 72 years old].

*“With the clinic in the leprosarium, we are always told there are no medicines when we visit, so that has made me not be consistent with visiting the hospital or clinic”* [Participant 10, male, 75 years old].

*“Doctors were not taking money initially, but now, anytime you visit the hospital, they take money from you. Also, you are not given medication for treatment if money has not been paid. They take 100 to 200 Ghana cedis for treatment”* [Participant 1, female, 86 years old].

*“I have decided not to go to the hospital again because I went and was not given any medication”* [Participant 19, male, 73 years old].

Physical Impairment captures the physical limitations experienced by participants as a result of the long-term effects of leprosy. Participants described conditions such as visual impairment and painful or wounded legs, which made mobility difficult and limited their ability to travel to health facilities independently. The following quotes summarise their responses,

*“I do not go to the hospital often.....Since I can no longer see or have the capacity to walk, I hardly access the hospital”* [Participant 18, female, unknown age].

*“I got infected in my childhood days; however, I got cured, but because of the wounds, I do not feel comfortable... Walking has been my greatest challenge in accessing health facilities”* [Participant 13, female, unknown age].

*“I do not go to the hospital often due to pain in my legs, and getting a vehicle is difficult. I cannot walk, and at times I just have to crawl before getting to the hospital”* [Participant 14, male, 63 years old].

Socio-economic Challenges (Direct and Indirect Costs) reflect the financial and livelihood-related difficulties that influenced participants’ health-seeking behaviour. These can be distinguished into direct costs, the out-of-pocket transport fares and medication or consultation fees that participants had to raise before a health facility visit could happen at all, and indirect costs, the loss of income from unemployment and the erosion of participants’ capacity to earn, which left them with little to draw on when a direct cost arose. The following quotes summarise their responses,

*“At first, I used to visit the hospital very often, but due to my current state, I no longer have the strength to do that nor have the money to do that....... The absence of money has been a major challenge for me......Was working initially, but due to my health condition, I am no longer doing anything”* [Participant 10, male, 75 years old].

*“My only difficulty is money, in that I would have to sell my fowls before I can get money to go seek medical treatment anytime I feel ill, and if I do not have it, I would have no option but to stay at home”* [Participant 17, female, 90 years old].

## Discussion

This qualitative descriptive study identified four themes among persons cured of leprosy in Godokpe, Ho Municipality: Knowledge, Social Treatment and Stigma, Health-Seeking Behaviour, and Challenges Influencing Health-Seeking.

Participants in this study predominantly attributed leprosy to supernatural forces, such as bewitchment, ancestral curses, or, in one case, a family-wide transmission through a cursed food item, with only one participant identifying a bacterial cause. This finding closely mirrors a qualitative study conducted in the same district, which similarly found that community members and persons affected by leprosy attributed the disease to supernatural forces rather than biomedical transmission [11]. A comparable pattern was reported among leprosy-affected individuals in urban rehabilitation centres in Ethiopia, where limited knowledge and attribution to supernatural causes or divine will were found to delay care-seeking and contribute to advanced disease presentation at diagnosis [21]. That this pattern recurs across three qualitative studies of affected populations in different countries, despite differences in setting and healthcare infrastructure, suggests supernatural causal attribution may function as a durable interpretive framework in leprosy-affected communities generally, rather than a gap in health education specific to Godokpe.

Although the comparison with a community-based survey in Nepal provides useful context, it has important limitations. In our study, biomedical awareness was nearly absent, with only one participant identifying bacteria as the cause, whereas in the Nepal study, 62.6% of respondents demonstrated at least partial recognition of a bacterial cause [22]. This disparity in proportions, coupled with differences in sample size, methodology, and population (general community members rather than PCLs specifically), cautions against direct equivalence. Moreover, the Nepal study employed a structured survey method, while our findings emerged from qualitative interviews, which may capture perceptions differently. Taken together, these factors suggest that while both settings reveal persistent misconceptions, the empirical alignment should be interpreted with caution rather than as a strict parallel.

Participants’ accounts of social treatment reveal a pattern distinct from the causal-belief: rather than uniform stigma, participants described two related but separable phenomena, one at the interpersonal level and one at the institutional level. At the interpersonal level, several participants recalled being isolated or avoided by family or community members out of fear of infection, particularly at the onset of their illness, with at least one participant describing this avoidance as ongoing rather than resolved. This mirrors findings from an earlier qualitative study in southern Ghana, which similarly found that persons affected by leprosy experienced stigma and discrimination from family, friends, and community members, and that many preferred remaining in a leprosarium-type setting over attempting reintegration, viewing it as a place of safety rather than confinement [23]. The persistence of this preference in the present study, decades after participants’ individual cures, is consistent with survey evidence from China showing that cured leprosy patients residing in leprosarium-type settings report different psychosocial outcomes from those who returned to live at home, suggesting that the choice to remain in a settlement like Godokpe reflects an ongoing calculation about social safety rather than simple institutional dependency [24].

At the institutional level, however, three participants independently described a documented shift in how PCLs were formally treated at health facilities, from segregated seating and prioritised or deferred treatment in the past to equal treatment alongside non-affected patients in the present. That three unrelated participants described the same institutional practice and its reversal suggests this reflects an actual change in facility-level norms over time, rather than three isolated perceptions. This pattern is consistent with a life-course study in western Nigeria, which found that stigmatisation of leprosy, while historically severe, has been actively counteracted over time by facilitators including improved treatment access and shifting community narratives [25], and with evidence from a cross-sectional study in Sri Lanka showing that experienced stigma, while still present, was weakly but significantly associated with longer time since diagnosis, consistent with stigma softening rather than remaining static over the course of a person’s life with the disease [26]. By contrast, a qualitative study of leprosy-affected women in Niger found that stigmatising treatment by healthcare providers, including isolation and refusal to treat, remained a current and ongoing barrier to care rather than a resolved historical practice [27]; the contrast between that finding and the institutional improvement described by participants in this study suggests that facility-level destigmatisation may be uneven across settings, and that Godokpe’s specific, long-standing relationship with the Ho polyclinic system may be a relevant factor rather than something to assume applies to PCLs generally.

Participants in this study did not rely on a single source of care, but navigated between biomedical facilities, herbal medicine, self-medication, and church-based prayer, drawing on different sources according to a perceived severity hierarchy: biomedical care as the default for serious conditions, and herbal, self-medicated, or spiritual options for milder symptoms or when biomedical care was inaccessible. This pattern of sequential rather than exclusive health-seeking has been documented elsewhere in leprosy-affected populations. A qualitative study in Madagascar found that most newly affected individuals initially consulted a trusted traditional healer before turning to biomedical or other providers recommended by relatives, only once initial treatment did not resolve their symptoms [28]. A community study in Nepal similarly found that a local medical shop was typically the first point of contact for skin symptoms, with belief in self-cure and reliance on traditional medicine contributing to delays before a formal leprosy diagnosis was reached [29]. Read alongside these findings, the pluralistic pattern in this study appears less like three competing belief systems and more like a common, sequential help-seeking logic in leprosy-affected communities, in which biomedical care is neither rejected nor automatically preferred, but arrived at conditionally.

This study’s findings on health-facility-level barriers are also consistent with broader determinants identified in the leprosy health-seeking literature. A qualitative study in Sierra Leone found that healthcare-seeking is shaped simultaneously at the individual, community, and healthcare-system level, with poor access, limited services, and economic motives at the system level compounding individual misconceptions and community-level stigmatisation [30]. Similarly, a qualitative study of leprosy retreatment patients in Brazil found that the quality of the relationship between healthcare professionals, patients, and carers was a decisive factor in whether patients trusted and adhered to treatment, with disorganised care and poor communication undermining engagement independently of patients’ own knowledge or beliefs [31]. This suggests that the barriers described by participants in the present study, particularly the drug unavailability and unexpected charges discussed below, are not incidental frustrations but a documented, system-level determinant of health-seeking behaviour in this population globally, not only in Godokpe.

One participant in this study described the church as her primary and preferred source of health-seeking, a pattern that, while representing only one of the twenty participants, is consistent with the broader role of religious belief as an active treatment-seeking pathway, rather than a passive fallback, that has been documented elsewhere in leprosy-affected communities in Ghana, where reliance on divine forms of healing was identified as one of several factors, alongside stigma and poor symptom recognition, shaping when and whether individuals sought biomedical care [11].

The informal charging for medication described by two participants in this study is consistent with a documented, system-wide pattern rather than an isolated grievance. A large facility-exit survey across three regions of Ghana found that 46.9% of insured National Health Insurance Scheme (NHIS) clients still made out-of-pocket payments for services nominally covered by the scheme, with drug unavailability at the facility accounting for 67.9% of such payments [32]. Read against this evidence, this finding is unlikely to reflect a Godokpe-specific failure, but rather a local instance of a broader, quantified gap between NHIS entitlement on paper and covered care in practice. This raises a question beyond individual health-seeking behaviour into rights and accountability: for a population such as PCLs, who may hold a specific entitlement to free leprosy-related care in addition to any general NHIS coverage, informal charging represents a compounded barrier warranting direct investigation into whether Godokpe residents’ entitlements are being honoured in practice, not only in policy. Taken together, these findings point to a policy-relevant gap: post-cure support for PCLs, distinct from active-case management, remains underdeveloped even where institutional destigmatisation has genuinely improved. Godokpe’s PCLs continue to face interpersonal stigma, informal charging for nominally free care, and structural barriers largely unaddressed by policies built around active leprosy treatment rather than long-term post-cure welfare. These findings align with SDG 1 (No Poverty), SDG 3 (Good Health and Well-Being), and SDG 10 (Reduced Inequalities), given the direct links between unemployment, financial hardship, and delayed care, and the documented gap between NHIS entitlement and covered care in practice.

## Conclusion

Across all three objectives of this study, findings trend in a consistent direction: cure did not resolve the non-biomedical framing of illness, the practical necessity of pluralistic care-seeking, or the layered vulnerability that together keep PCLs at the margins of the health system decades after treatment ends. This suggests that leprosy’s post-cure burden in settings like Godokpe is not primarily a residual knowledge gap to be corrected, but a durable social and economic position that biomedical cure alone does not change.

This study adds two findings not clearly established in the existing literature on this population. First, institutional and interpersonal stigma appear to follow different trajectories, with facility-level treatment of PCLs showing genuine improvement over time while family- and community-level avoidance persists unevenly; prior work on leprosy stigma has generally not distinguished between these two levels. Second, the informal charging for medication described independently by two participants, situated against national evidence of widespread out-of-pocket payment by insured NHIS clients, raises a specific accountability question: whether PCLs’ entitlements to free care are being honoured in practice, which has not been directly examined in prior post-cure health-seeking research.

These findings imply that post-cure support for PCLs requires attention distinct from, and in addition to, active-case detection and stigma-awareness messaging: specifically, verification of fee-exemption entitlements at the facility level, and sustained family- and community-level engagement where institutional change has not yet reached. Future research should extend beyond this single site to establish whether the institutional-improvement and informal-charging patterns identified here recur in other PCL communities in Ghana, incorporate the perspectives of healthcare providers and family members alongside those of PCLs, and track health-seeking longitudinally rather than at a single point in participants’ post-cure lives.

### Strengths and limitations

This study’s qualitative descriptive design stands as a core strength, allowing a comprehensive, data-rich account of how persons cured of leprosy understand and navigate health-seeking in a historically stigmatised setting. By conducting interviews in the familiar environment of Godokpe, the research built trust and elicited candid, detailed narratives rarely captured in quantitative work. Purposive sampling to saturation, combined with Atlas.ti-supported systematic coding ensured analytical transparency and auditability. The use of verbatim transcription, cross-checked translation from Ewe to English, and iterative theme refinement further strengthened trustworthiness. COREQ adherence enhanced reporting rigour, while the Ho-specific focus builds directly on prior local studies, offering fresh insight into post-cure realities.

Limitations include the small, purposively selected sample of 20 participants from a single site, which restricts generalizability beyond similar segregated communities. The older age and long-term residence of most participants may have skewed responses toward entrenched beliefs or resignation, potentially differing from those of younger or urban PCLs. Self-reported accounts carry recall bias risk, especially for distant events. Translation processes, though verified, could introduce subtle interpretive shifts. The cross-sectional design provides only a snapshot, limiting insight into how health-seeking evolves. Interviews lasted approximately 20–30 minutes; while this duration was judged appropriate given participants’ age and the risk of fatigue, it is shorter than is typical for studies aiming at the fullest possible depth of engagement, and may have constrained the elaboration of some accounts.

Methodologically, the second researcher’s independent coding review covered a subset of transcripts rather than the full dataset, and no prior relationship with participants was established before the study began; beyond this, no repeat interviews were carried out to explore the consistency of accounts over time. No member-checking or return of themes to participants was conducted, and no repeat interviews were carried out to explore the consistency of accounts over time; both would have strengthened confidence in the interpretations offered.

## Supporting information

S1 File Completed COREQ (Consolidated Criteria for Reporting Qualitative Research) checklist detailing the reporting of the research team, study design, and analysis for this study, cross-referenced to the relevant sections of the manuscript. (DOCX)

S2 File The ethics-approved semi-structured interview guide used for in-depth interviews with participants, including the introductory consent script read to participants before each interview, organised into four domains: knowledge and perceived causes of leprosy, social treatment and experiences of stigma, current health-seeking behaviour, and retrospective experience around initial diagnosis. (DOCX)

## Abbreviations

COREQ: Consolidated Criteria for Reporting Qualitative Research
GCP: Good Clinical Practice
G2D: Grade-2 Disabilities
IDI: In-Depth Interview
ILEP: International Federation of Anti-Leprosy Associations
LEAP: Livelihood Empowerment Against Poverty
MDT: Multidrug Treatment
NGO: Non-Governmental Organization
NTDs: Neglected Tropical Diseases
PCLs: Persons Cured of Leprosy
PEP4LEP: Post-Exposure Prophylaxis for Leprosy
QoL: Quality of Life
SDGs: Sustainable Development Goals
WHO: World Health Organization
