# Supplementary material for: Understanding influences on health-seeking behaviours among persons cured of leprosy in the Godokpe community, Volta Region of Ghana: A qualitative descriptive study
Source: PLoS Negl Trop Dis. 2026 Sep 30;20(9):e0014753. doi: 10.1371/journal.pntd.0014753 (PMC13626315; doi:10.1371/journal.pntd.0014753)
Supplement: Supplementary file 1 [file pntd.0014753.s001.docx]

Reporting adhered to the Consolidated Criteria for Reporting Qualitative Research (COREQ)

| **No.** | **Item** | **Guide Questions/Description** | **Response from Manuscript** |
| --- | --- | --- | --- |
| **Domain 1: Research Team and Reflexivity** |  |  |  |
| 1 | Interviewer/facilitator | Which author(s) conducted the interview or focus group? | Two trained research assistants conducted the face-to-face interviews. |
| 2 | Credentials | What were the researcher’s credentials? | Authors were affiliated with the University of Health and Allied, Sciences and Kwame Nkrumah University of Science and Technology. |
| 3 | Occupation | What was their occupation at the time of the study? | Researchers were academics/public health researchers. |
| 4 | Gender | Was the researcher male or female? | Not reported. |
| 5 | Experience and training | What experience or training did the researcher have? | Research assistants were trained before data collection. |
| 6 | Relationship established | Was a relationship established prior to study commencement? | Not reported. |
| 7 | Participant knowledge of the interviewer | What did the participants know about the researcher? | Participants were informed about the purpose of the study before participation. |
| 8 | Interviewer characteristics | What characteristics were reported about the interviewer/facilitator? | Not reported. |
| **Domain 2: Study Design** |  |  |  |
| 9 | Methodological orientation and theory | What methodological orientation underpinned the study? | Phenomenological qualitative design. |
| 10 | Sampling | How were participants selected? | Purposive sampling. |
| 11 | Method of approach | How were participants approached? | Face-to-face approach and interviews. |
| 12 | Sample size | How many participants were in the study? | Twenty participants. |
| 13 | Non-participation | How many people refused to participate or dropped out? | Not reported. |
| **Setting** |  |  |  |
| 14 | Setting of data collection | Where was the data collected? | Cured Lepers’ Village (CLV), Ho Municipality, Volta Region, Ghana. |
| 15 | Presence of non-participants | Was anyone else present besides the participants and researchers? | Not reported. |
| 16 | Description of sample | What are the important characteristics of the sample? | Socio-demographic characteristics were presented in Table 1. |
| **Data Collection** |  |  |  |
| 17 | Interview guide | Were questions/prompts provided? Was it pilot tested? | Semi-structured interview guide was used and pilot tested. |
| 18 | Repeat interviews | Were repeat interviews carried out? | Not reported. |
| 19 | Audio/visual recording | Did the research use audio or visual recording? | Interviews were audio-recorded. |
| 20 | Field notes | Were field notes made? | Not reported. |
| 21 | Duration | What was the duration of the interviews? | Approximately 20–30 minutes. |
| 22 | Data saturation | Was data saturation discussed? | Yes, recruitment continued until thematic saturation was reached. |
| 23 | Transcripts returned | Were transcripts returned to participants? | Not reported. |
| **Domain 3: Analysis and Findings** |  |  |  |
| 24 | Number of data coders | How many data coders coded the data? | Initial coding by researchers with independent review by a second researcher. |
| 25 | Description of the coding tree | Did authors provide a description of the coding tree? | Themes and subthemes were presented in Table 2. |
| 26 | Derivation of themes | Were themes identified in advance or derived from the data? | Themes were derived inductively from the data. |
| 27 | Software | What software was used to manage the data? | Atlas.ti version 7.5.0. |
| 28 | Participant checking | Did participants provide feedback on the findings? | Not reported. |
| **Reporting** |  |  |  |
| 29 | Quotations presented | Were participant quotations presented? | Yes, quotations with participant identifiers were included. |
| 30 | Data and findings consistent | Was there consistency between the data and findings? | Yes. |
| 31 | Clarity of major themes | Were major themes clearly presented? | Yes, major themes were clearly presented. |
| 32 | Clarity of minor themes | Is there a description of diverse cases or minor themes? | Yes, variations in beliefs and healthcare practices were discussed. |
