# Supplementary material for: Understanding influences on health-seeking behaviours among persons cured of leprosy in the Godokpe community, Volta Region of Ghana: A qualitative descriptive study
Source: PLoS Negl Trop Dis. 2026 Sep 30;20(9):e0014753. doi: 10.1371/journal.pntd.0014753 (PMC13626315; doi:10.1371/journal.pntd.0014753)
Supplement: Supplementary file 2 [file pntd.0014753.s002.docx]

**Understanding influences on health-seeking behaviours among persons cured of leprosy in Godokpe (the Cured Lepers’ Village), Ho Municipality, Volta Region of Ghana: A qualitative descriptive study**

**In-depth Interview Guide**

Good morning/afternoon. My name is [interviewer’s name], and I am part of a research team from Fred N. Binka School of Public Health, University of Health and Allied Sciences, conducting a study on the health-seeking experiences of persons cured of leprosy living in Godokpe. The purpose of this study is to understand how you, and others who have been cured of leprosy, think about health, where you go for care when you are unwell, and any difficulties you may face in getting the care you need. We hope that what we learn will help inform how services and support are provided to persons cured of leprosy in the future.

Before we begin, I want to explain a few things about your participation:

- Your participation in this interview is completely voluntary. You do not have to answer any question you do not wish to answer, and you may stop the interview at any time, for any reason, without any negative consequence to you or the support you receive here.
- Everything you share with us will be kept confidential. Your name will not be used in anything we write or publish; instead, we will refer to you only by a number. We will also be careful not to include other details that could allow someone to identify you.
- With your permission, I would like to audio-record our conversation so that I can listen carefully to you now rather than spend the time writing, and so that I can accurately capture what you tell me afterwards. The recording will only be heard by the research team and will be kept securely. May I have your permission to record?
- This interview will take approximately 20 to 30 minutes. If at any point you would like to rest or stop, please let me know.
- There are no right or wrong answers. We are simply interested in your own experiences and your own views.

**SOCIO-DEMOGRAPHIC**

| **NO.** | **QUESTIONS** | **RESPONSE** |
| --- | --- | --- |
| 1 | AGE |  |
| 2 | Marital status | 1. Married 2. Single 3. Cohabitation 4. Divorce/ Separated |
| 3 | Educational Level | 1. Tertiary 2. Secondary 3. JHS 4. Primary 5. None |
| 4 | What is your occupation | 1. Student 2. Farming 3. Trading 4. Fishing 5. Teaching 6. Other specify……. |
| 5 | Religion | 1. Christianity 2. Islam 3. Traditional 4. Others Specify……. |

**PERSONS CURED OF LEPROSY INTERVIEW GUIDE**

6. What brought you to the leprosarium?

Probe:

How old were you at the time, and who decided for you to come here?

Did you consider any other options before coming to the leprosarium?

7. How does one get Leprosy?

Probe:

Where did you first hear this explanation from: family, community, or somewhere else?

Has your understanding of the cause changed since then? If so, what changed it?

8. How are you treated by society?

Probe:

Has the way society treats you changed over time? Can you describe how it was before compared to now?

Can you give a specific example of a time you felt well treated, and a time you felt poorly treated?

9. How are you treated by your family?

Probe:

Has your relationship with your family changed since you were cured?

Is there a difference in how different family members (e.g., parents versus children/grandchildren) have treated you?

10. What are some of the difficulties you go through when accessing public institutions? (School/ Hospital/ Bank/ other offices)

Probe:

When you say [money/transport] is a difficulty, can you walk me through what that actually costs you, both the direct cost of the visit and anything else you lose by going (e.g., time, missed income)?

Is there a specific institution that has been more difficult than others?

11. How are you treated by Nurses and Administrators in the leprosarium?

Probe:

Has this treatment changed over the years you’ve been here? Can you describe what it was like when you first arrived compared to now?

Is there a difference between how you’re treated here versus at outside facilities?

12. Do you feel treated fairly like others who have never been affected by leprosy when you visit hospitals outside the village?

Probe:

Was this always the case, or has it changed over time? If it changed, when did you first notice the difference?

Can you describe what happens when you sit and wait to be seen? Is it different from how it used to be?

13. In your view, how do you want persons affected by leprosy to be treated in Ghana?

Probe:

What would that treatment look like in practice from the government, from health facilities, from the community?

Is there something specific that has happened to you that you’d want to see change for others?

14. Where do you go or whom do you consult when you are ill?

Probe:

Does where you go depend on how serious you feel the illness is? Can you walk me through how you decide?

Is this different from where you would have gone five or ten years ago?

15. How often do you access the hospital?

Probe:

Has this frequency changed recently, and if so, what changed it?

16. What factors prevent you from accessing the hospital?

Probe:

When you say money is a factor, is it the cost of the visit itself, the cost of getting there, or both?

Has there been a specific incident that made you less willing to go?

17. Where do you normally seek health care from?

Probe:

What makes you choose that place over other available options?

If that option weren’t available, where would you go instead?

18. How long did it take you to visit the hospital when you had symptoms of leprosy?

Probe:

What were you doing or trying instead during that time before you went?

Looking back, what would have made you go sooner?

19. What did you think when you noticed those symptoms?

Probe:

Did you discuss it with anyone at the time? What did they say?

20. What did you do about the symptoms?

Probe:

Why did you choose that particular response first, before anything else?

21. Do you have any other alternatives for your health?

Probe:

When do you use that alternative instead of the hospital? Is it about cost, distance, severity, or something else?

22. What has been your experience living in the community?

Probe:

How has that experience changed since you first arrived?

Is there anything about community life here that you would want people outside to understand better?
