# Supplementary material for: Understanding influences on health-seeking behaviours among persons cured of leprosy in the Godokpe community, Volta Region of Ghana: A qualitative descriptive study
Source: PLoS Negl Trop Dis. 2026 Sep 30;20(9):e0014753. doi: 10.1371/journal.pntd.0014753 (PMC13626315; doi:10.1371/journal.pntd.0014753)
Supplement: Supplementary file 3 [file pntd.0014753.s003.docx]

**General Reflection.**

**Although the study trying to bridge the knowledge gap in the heath seeking behaviors of persons cured of leprosy, the findings are more or less similar with studies of patients on treatment.**

**While trying to make the writing more word economic, some important ideas were lost. Consider re-read and writ it.**

**The discussion mostly focused on repeating the result and comparing with other studies without explaining the underlying notions.**

**Introduction**

- In the second paragraph of the introduction lacks coherence, it jumps from new case to poverty and disability. Furthermore, I didn't get the point how PEP4LEP activity in the paragraph. Hence, which needs revision
- From line number 103 to 105 use one of the data from WHO 2024 global leprosy update, which causes confusion for readers.
- In the third paragraph, line number 112, the transition from epidemiologic information to the next lacks clear transitions. revise the paragraph properly.
- On line number 130, do we use the term “Lepers” these days?
- Your study aimed to investigate about health seeking behaviors of persons affected by leprosy post MDT. How does “Globally, the 5.3% G2D rate among 2024 new cases signals systemic delays in intervention” supports your arguments?

**Methodology**

- In line 153 under study setting, the term *“Leper”* is not currently used terminology and in some contexts, it is considered us derogatory. In addition to this section, it has been indicated throughout the paper. Replace it with appropriate term.
- Although the study setting contains many important points that describes where the study conducted, it also includes many unnecessary details. Try to condense it focusing the where the study was conducted and how it’s ideal place the intended study.
- Where did you conduct the interview?
- In the study population, did you include newly treatment completed or older cases? Both groups could have different level of knowledge, health care sources and perceived challenges of health care seeking behaviors. Furthermore, after MDT completion, one of the major reasons for frequent health care seeking depends on development of reaction, existence of disability and so on. Hence better to characterize participants included in the study.
- In the inclusion and exclusion criteria, take statements that talks about ethics related issues into dedicated ethics section.
- “Cured Lepre Foundation” is it a patient association? If so, does they have ethics reviewing committee?
- In the trustworthiness section, since you involved large number of older people cured of leprosy, they could have memory limitation. Could you elaborate more about confirmability, how truly your interpretation is grounded in the participants lived experience?

**Result**

- One of the good things in this study is involvement of large number of female participants. Global epidemiologic data indicates that males are more vulnerable than women and the case load is also disproportionately higher among male patients. Why this gender discrepancy happened, not yet well explained. Furthermore, the health seeking behavior of both male and female could be influenced by distinct factors. So, batter to take into consideration in your interpretation of the data.
- Within your result “Knowledge” is one of the themes identified from the data. This theme sub-themed as General Cause and Sign and symptom. In the general cause, one of the supporting quotes relates the disease with dietary practice. “After eating yam” how this could be a superstation belief?
- In the health seeking theme, I got confused. Are we aiming to study the current health seeking behavior influencers or before diagnosis and treatment with MDT? Because the either the result or the quote indicates a current practice or before the diagnosis leprosy.
- In line number 348, a paragraph about herbal medicine indicated as an alternative pathway to seek care. However, according the participants account, herbal medicine was described as complemental to modern biomedical approach. Thus, rephrase your statements as complemental rather than as independent alternative path. Unless it could raise the issue of confirmability on your interpretation.
- One of the key features in phenomenological study is putting aside our previous perception called bracketing. The statement below indicates that the interpretation is based on your life experience. Hence, good to reflect your role and perspective.
  - *“self-medication is common phenomenon is among Ghanaians, especially when the health conditions are deemed to be mild and not needing expert attention”*
- The health seeking challenge’s sub-theme, access barrier (distance and transportation), the service gap (unavailability of drug), and behavioral impact (due to lack of trust and reduced motivation due to unavailability of the drug). In general, rather than health seeking challenge, this sub-theme could be structural barriers to health seeking. Re-read the transcript and consider re-thematization of the data.
- The subtheme physical challenge bit confusing for the reader. I recommend to make it physical impairment as a challenge for health care seeking.
- To make socio-economic sub-theme more meaningful, better to classify it as direct cost, indirect cost and so on. So that we can easily learn which socio-economic impact has the largest impact on health seeking behavior. Furthermore, it would help you to draw meaning recommendation.
- In the result section, from line number 326-328, under the subtheme of health seeking behavior, most of the participants sought health service from health facilities. However, you have mentioned orthodox health care service, is it separate health facility or health service under the orthodox cure in addition to spiritual service? please make the paragraph clear for reader.

**Discussion**

- Although the comparison with Nepal provides useful context, it has important limitations. In our study, biomedical awareness was nearly absent, with only one participant identifying bacteria as the cause, whereas in Nepal 62.6% of respondents demonstrated at least partial recognition. This disparity in proportions, coupled with differences in sample size, methodology, and cultural context, cautions against direct equivalence. Moreover, the Nepal study employed structured survey methods, while our findings emerged from qualitative interviews, which may capture perceptions differently. Taken together, these factors suggest that while both settings reveal persistent misconceptions, the empirical alignment should be interpreted with caution rather than as a strict parallel.
- When you compare your findings with other empirical evidences, take into account the measurement and sample size differences could have its impact. So, the interpretation must be cautiously.
- Please cite reference for this conclusive statement…

*“Yet, the Ho Leprosarium residents, many isolated for decades post-cure, showed even deeper entrenchment in spiritual explanations, perhaps because prolonged stigma reinforces cultural narratives as a coping mechanism”*

- From line 467 to 470, the paragraph attempts to integrate findings from India, Nepal and you study with a single sentence, which reduces clarity for the reader. Consider to improve the sentence.
- Result statements are repeated through our various sections of the discussion. Rather focus on the interpretations of the result.

**Conclusion**

- The conclusion is far beyond the scope of the study. Overstated conclusions should be rewritten.
- Some unrealistic statements like “WHO may not be engage and shift budget for economic empowerment” should be rewritten.

**Strength and limitation**

- Despite the paragraphs crafted well, some methodologic related and interpretation related limitations were not completely outlined.
