# Supplementary material for: Understanding influences on health-seeking behaviours among persons cured of leprosy in the Godokpe community, Volta Region of Ghana: A qualitative descriptive study
Source: PLoS Negl Trop Dis. 2026 Sep 30;20(9):e0014753. doi: 10.1371/journal.pntd.0014753 (PMC13626315; doi:10.1371/journal.pntd.0014753)
Supplement: Supplementary file 4 [file pntd.0014753.s005.docx]

# Response to Reviewers

**Manuscript: Understanding influences on health-seeking behaviours among persons cured of leprosy in Godokpe (the Cured Lepers’ Village), Ho Municipality, Volta Region of Ghana: A qualitative descriptive study**

Dear Editor,

We thank the Editor and the three reviewers for their thorough and constructive evaluation of our manuscript. We have carefully considered every comment and revised the manuscript extensively in response, including a reframing of the study design from phenomenological to qualitative descriptive (with reflexive thematic analysis), a substantial rebuild of the Discussion section, the addition of a fourth Results theme (Social Treatment and Stigma) grounded in participant transcripts, and terminology, ethics, and reporting revisions throughout. Below we address each reviewer’s comments in turn, indicating the corresponding revision in the manuscript.

## Response to Reviewer #1

### General Reflection

**Comment:** The findings are more or less similar to studies of patients on treatment.

**Response:** We agree that this comparison needed to be made explicit rather than left implicit. In the revised Discussion, we now directly situate our findings against studies of both currently affected and cured populations (e.g., Rasolofozafy et al., 2025; Subedi & Engelbrektsson, 2018), explaining where the patterns align and where our post-cure focus reveals something distinct, particularly the informal-charging and institutional-stigma-shift findings, which are not addressed in studies of patients still on treatment.

**Comment:** While trying to make the writing more word economic, some important ideas were lost. Consider re-read and write it.

**Response:** We have restored analytical depth throughout Results and Discussion, particularly around Participant 3’s occupational explanation for his biomedical knowledge, the temporal/institutional dimension of stigma, and the free-medication finding, rather than presenting these only as brief, under-explained observations.

**Comment:** The discussion mostly focused on repeating the result and comparing with other studies without explaining the underlying notions.

**Response:** The Discussion has been substantially rebuilt. Each theme now opens with an interpretive claim rather than a restatement of Results, and comparisons to other studies are used to support or contextualise that interpretation rather than stand alone.

### Introduction

**Comment:** The second paragraph of the introduction lacks coherence, it jumps from new case to poverty and disability. Furthermore, I didn’t get the point how PEP4LEP activity in the paragraph. Hence, which needs revision.

**Response:** Revised. PEP4LEP is now explicitly framed as a regional response to the delayed-diagnosis problem described in the preceding sentence, rather than an unconnected data point.

**Comment:** From line number 103 to 105 use one of the data from WHO 2024 global leprosy update, which causes confusion for readers.

**Response:** The Ghana statistics paragraph has been streamlined to the two figures directly relevant to the paragraph’s argument (278 new cases; 15.1% G2D rate), removing redundant per-population decimal rates, child-case figures, and gender breakdown.

**Comment:** In the third paragraph, line number 112, the transition from epidemiologic information to the next lacks clear transitions. Revise the paragraph properly.

**Response:** An explicit bridging sentence has been added connecting the Volta Region case-trend data to the description of the Ho Leprosarium and Godokpe.

**Comment:** On line number 130, do we use the term “Lepers” these days?

**Response:** “Cured Lepers’ Village” has been replaced with “Godokpe” as the primary term throughout the manuscript, consistent with person-first language.

**Comment:** How does “Globally, the 5.3% G2D rate among 2024 new cases signals systemic delays in intervention” support your arguments?

**Response:** We agree that this statistic, which concerns new-case detection delay, did not support the paragraph’s argument about post-cure support gaps. The sentence has been removed.

### Methodology

**Comment:** The term “Leper” is not currently used terminology and in some contexts, it is considered as derogatory. Replace it with appropriate term.

**Response:** Addressed throughout, as above.

**Comment:** The study setting also includes many unnecessary details. Try to condense it, focusing on where the study was conducted and how it is an ideal place for the intended study.

**Response:** The Study Setting section has been condensed while retaining the historical detail necessary to justify the site’s relevance to the study.

**Comment:** Where did you conduct the interview?

**Response:** Now stated explicitly: interviews were conducted in person, in private spaces within Godokpe.

**Comment:** In the study population, did you include newly treatment completed or older cases? Better to characterize participants included in the study.

**Response:** A sentence has been added to the Study Population confirming that all 20 participants were long-term cured residents (18+ years), rather than recently treated individuals.

**Comment:** In the inclusion and exclusion criteria, take statements that talk about ethics related issues into dedicated ethics section.

**Response:** Consent and voluntary-withdrawal language has been moved from Inclusion/Exclusion Criteria into Ethics Considerations.

**Comment:** “Cured Lepre Foundation” is it a patient association? If so, does they have ethics reviewing committee?

**Response:** Clarified in Ethics Considerations: the Cured Lepers Foundation is a formally recognised non-governmental organisation with a community-access and welfare-administration role; it does not hold, and was not treated as holding, ethics-review authority. UHAS-REC approval constituted the study’s binding ethical authorisation.

**Comment:** Could you elaborate more about confirmability, how truly your interpretation is grounded in the participants’ lived experience?

**Response:** The Confirmability section has been expanded to address memory limitations in an elderly sample explicitly, and a new Reflexivity subsection has been added.

### Result

**Comment:** Involvement of large number of female participants... why this gender discrepancy happened, not yet well explained.

**Response:** A new paragraph following Table 1 addresses the gender imbalance, situating it in the demographic and social history of Godokpe rather than treating it as unexplained.

**Comment:** “After eating yam” how this could be a superstition belief?

**Response:** A clarifying paragraph now explains the mystical rather than biomedical logic underlying this account, distinguishing it from a literal food-contamination claim.

**Comment:** Are we aiming to study the current health seeking behavior influencers or before diagnosis and treatment with MDT?

**Response:** The Health-Seeking Behaviour section now explicitly states that it describes participants’ current, ongoing practices, distinct from the historical diagnosis process.

**Comment:** Herbal medicine was described as complemental to modern biomedical approach... rephrase your statements as complemental rather than as independent alternative path.

**Response:** Revised throughout, herbal medicine is now consistently framed as complementary rather than alternative, consistent with participants’ own accounts.

**Comment:** Good to reflect your role and perspective (re: bracketing, the self-medication statement).

**Response:** The sentence attributing self-medication to a general assumption about Ghanaian behaviour has been removed and replaced with an explanation grounded directly in participants’ stated reasons (cost, drug unavailability, loss of trust).

**Comment:** This sub-theme could be structural barriers to health seeking. Re-read the transcript and consider re-thematization.

**Response:** Renamed to “Structural Barriers to Health-Seeking,” now explicitly organised into access barrier and service gap components.

**Comment:** Make it physical impairment as a challenge for health care seeking.

**Response:** Renamed to “Physical Impairment.”

**Comment:** Classify socio-economic sub-theme as direct cost, indirect cost, and so on.

**Response:** Renamed to “Socio-economic Challenges (Direct and Indirect Costs),” with the paragraph now explicitly distinguishing the two.

**Comment:** You have mentioned orthodox health care service, is it separate health facility... please make the paragraph clear for reader.

**Response:** Clarified: “orthodox” refers to conventional biomedical facilities (Ho Polyclinic, Ho Teaching Hospital), as distinct from herbal or spiritual sources.

### Discussion

**Comment:** The Nepal comparison has important limitations; measurement and sample size differences should be interpreted cautiously; please cite reference for the stigma/coping-mechanism claim; lines 467-470 reduce clarity; result statements are repeated rather than interpreted.

**Response:** The Discussion has been substantially rebuilt. We have retained and applied the reviewer’s own caution regarding the Nepal comparison nearly verbatim. The unsupported claim regarding stigma reinforcing cultural narratives has been removed. Dense multi-study sentences have been broken up, and each theme now opens with interpretation rather than a restatement of Results.

### Conclusion

**Comment:** The conclusion is far beyond the scope of the study. Overstated conclusions should be rewritten. “WHO may not be engage and shift budget for economic empowerment” should be rewritten.

**Response:** The Conclusion has been rebuilt to state findings’ direction relative to the study’s objectives, articulate the study’s specific contribution to existing knowledge, and offer implications scoped to what a single-site qualitative study can support. The WHO/ILEP funding-expansion recommendation has been removed.

### Strength and limitation

**Comment:** Some methodologic related and interpretation related limitations were not completely outlined.

**Response:** The Limitations paragraph now additionally addresses: the partial (subset-only) second-coder review, incomplete documentation of researcher positionality, and the absence of member-checking or repeat interviews.

## Response to Reviewer #2

### General Comments

**Comment:** There appears to be some mismatch between the claimed design and the analysis as presented. Naming the specific tradition... would help. Alternatively, the authors might consider reframing the study as a qualitative descriptive design.

**Response:** We have reframed the study as a qualitative descriptive design using reflexive thematic analysis (Braun & Clarke, 2019), which better reflects the analysis as actually conducted. This is now reflected consistently in the Title, Abstract, Study Design, and Data Analysis sections.

**Comment:** The objectives as currently stated read as descriptive aims.

**Response:** Consistent with the design reframe above, the objectives are now presented as appropriate to a qualitative descriptive study rather than retrofitted to a phenomenological framing they did not match.

**Comment:** The interview duration appears relatively brief... the authors may wish to acknowledge and briefly justify this duration.

**Response:** The Data Collection Instrument and Procedure section now explicitly justifies the 20–30 minute duration on two grounds: fit with a descriptive rather than exhaustive-exploratory aim, and minimising fatigue among older participants. This is additionally now named as a limitation in Strengths and Limitations.

**Comment:** Several participants have unknown ages. Providing estimated ranges... would be a helpful addition.

**Response:** We reviewed our transcripts and records and found no reliable basis for estimating ranges for the five participants who could not recall their age. Table 1 and the accompanying text now state this explicitly, rather than presenting an unsubstantiated estimate.

**Comment:** Including WHO Disability Grades in the participant table, if/where available.

**Response:** WHO Disability Grades were not collected as part of this study’s data and are therefore not available for inclusion.

**Comment:** Authors should include a reflexivity statement... describe their positionality, disciplinary backgrounds, and relationship to the community.

**Response:** A new Reflexivity subsection has been added to Methods.

### Results

**Comment:** The analysis remains descriptive rather than interpretive. A striking analytical opportunity is missed with Participant 3.

**Response:** Participant 3’s biomedical fluency is now explicitly interpreted in relation to his occupational history as a former orderly at the leprosarium, offering a grounded explanation rather than leaving the outlier unaddressed.

**Comment:** The quotations included offer useful illustrations... could be strengthened further by incorporating slightly longer extracts.

**Response:** Having reviewed the source transcripts directly, we found that several existing quotations required correction for accuracy rather than extension. We have prioritised quotation accuracy over length, and retained the fullest verbatim material available for each illustrative quote.

**Comment:** The Health-Seeking Sources findings describe where participants go for care but not how they navigate between systems or what meanings these choices hold.

**Response:** The Health-Seeking Behaviour section now includes an interpretive paragraph proposing that participants navigate sources according to a perceived severity hierarchy, rather than presenting the sources as a simple list.

### Conclusions

**Comment:** The claim that the study “breaks new ground by centring phenomenological voices” might overstate the contribution.

**Response:** This claim has been removed, consistent with the design reframe.

**Comment:** The limitations could discuss the brevity of interviews.

**Response:** Added, as noted above.

**Comment:** One participant describes being denied free medications despite an apparent policy of free care, which raises questions about rights and accountability that go unexamined.

**Response:** This is now developed as a dedicated paragraph in Discussion, strengthened by a second, independently corroborating participant account, and situated against national evidence of informal charging among insured NHIS clients in Ghana (Akweongo et al., 2021), which suggests this reflects a broader documented pattern rather than an isolated grievance.

### Writing and Terminology

**Comment:** The term “lepers” (line 186) must be replaced with person-first language. The repeated use of “Cured Lepers’ Village” could be reconsidered; adopting the local name “Godokpe.” Consider deductive disclosure risk. Soften “predicament” (line 425).

**Response:** “Lepers” has been replaced with person-first language throughout. “Godokpe” is now used as the primary place name, with “Cured Lepers’ Village” retained only as a first-mention historical gloss. A statement addressing deductive disclosure has been added to Ethics Considerations. We have reviewed the manuscript for similarly-toned phrasing and revised accordingly.

## Response to Reviewer #3

### General Comments

**Comment:** The authors have stated a general aim for the study supported by 3 specific objectives, the last of which suggests they believe that challenges may be affecting health-seeking negatively - this does not however qualify a clear testable hypothesis. While the qualitative design is appropriate to address the stated objectives, the earlier point on hypothesis and framing of the 3rd specific objectives suggest some bias. How did the authors ensure that positive factors influencing health-seeking were not ignored?

**Response:** We agree that the original wording of the third objective, framed solely around "challenges," risked signalling a deficit-only orientation from the outset. We have revised the objective to read "examine the facilitators and challenges influencing their health-seeking behaviours" (Introduction), with corresponding updates to the Abstract for consistency. This is not merely a wording change: the analysis had, in fact, already surfaced facilitating factors, including a documented institutional shift toward equal treatment of PCLs at health facilities over time, and instances of continued family support, which are now presented under the new Social Treatment and Stigma theme. The revised objective wording brings the stated aim into alignment with what the study's findings and analysis actually capture.

**Comment:** It will be helpful to have more detailed description of the elements of their data collection instrument.

**Response:** The Data Collection Instrument and Procedure section now describes the interview guide’s four domains (knowledge/causes, social treatment and stigma, current health-seeking, and retrospective diagnosis-era experience) with representative example questions for each.

**Comment:** Include the specific ethics-approved IDI question guide as an attachment with their response.

**Response:** The ethics-approved interview guide will be included as a supplementary attachment with this resubmission.

**Comment:** Is the Cured Leper Foundation a formal registered entity and is there a legal mandate granting them oversight of free citizens cured of leprosy? Would it not be more appropriate to have a public authority approached for approval?

**Response:** We have clarified in Ethics Considerations that the Cured Lepers Foundation is a formally recognised non-governmental organisation whose role in this study was limited to community-level access and courtesy permission, not ethics oversight. The study’s binding ethical authorisation was obtained from the University of Health and Allied Sciences Ethics Review Committee (UHAS-REC), a recognised public ethics authority, as stated in the manuscript.

### Results

**Comment:** Table 1... doesn’t have information on educational background, although the discussion points to most of the study population having minimal formal education.

**Response:** Educational background was not systematically collected as part of this study’s structured socio-demographic data. Table 1 and the accompanying text now state this explicitly. We have additionally reviewed the Discussion to ensure it does not assert an educational-attainment claim for the population that our data cannot support.

**Comment:** The repeated use of “thus” in lines 261-263 suggests link to a pre-existing condition satisfied and confuses clear meaning.

**Response:** Corrected.

### Discussion

**Comment:** Avoid drawing weak linkage such as comparing knowledge/perceptions of healthcare workers to persons with a lived experience of disease, and desist from introducing author inferences and opinions which can raise questions on doubts.

**Response:** The Discussion has been substantially rebuilt. Comparisons to studies of healthcare workers, medical trainees, and general urban populations have been removed and replaced with comparisons to studies of leprosy-affected and cured populations specifically, which are more directly comparable to our study population.

**Comment:** Lines 467-470: Does Gopalakrishnan et al. assess KAP amongst persons with a lived experience of the disease or urban adults generally? The sentence structure appears to combine two ideas without punctuation.

**Response:** The Gopalakrishnan et al. comparison has been removed. The relevant paragraph now compares our findings to qualitative studies of leprosy-affected populations in Ghana and Ethiopia instead.

**Comment:** Lines 475-477: Contrasting the knowledge of medical trainees with interns is more likely the impact of medical education rather than patient-provider interaction. How do the authors know hospital interactions are/were likely rare?

**Response:** This comparison has been removed in its entirety, along with the unsupported claim regarding the rarity of hospital interactions.

**Comment:** Lines 490-494: The reader may struggle to find why the finding of one woman preferring the church is being compared to a study where traditional healers gave way to biomedical care only if ineffective; the explanatory sentence is also too long.

**Response:** We agree that this comparison was not well matched. The church-preference finding is now discussed in relation to a Ghana-specific study on divine healing as an active care-seeking pathway (Ahiabu & Tabong, 2024), rather than the Madagascar timing-sequence study, which is used elsewhere in the Discussion for a more appropriate purpose (the sequencing of care-seeking sources generally).

**Comment:** The authors are advised to overhaul the discussion section.

**Response:** The Discussion has been rebuilt from its literature base upward, organised thematically (Knowledge, Social Treatment and Stigma, Health-Seeking Pluralism, Barriers and Accountability), with each section opening on interpretation rather than a restatement of Results, and comparisons restricted to studies of comparable populations wherever possible.

### Conclusions

**Comment:** The discussion section should be further tightened... overhaul to more appropriately compare findings in this specific population with similar populations (PCLs) from other studies in other geographic regions, or with other cured patient populations.

**Response:** The rebuilt Discussion now draws primarily on studies of PCLs and leprosy-affected populations across Nigeria, Niger, Sri Lanka, China, Malaysia, Ethiopia, Sierra Leone, and Madagascar, rather than the more distant comparison populations used previously.

### Writing

**Comment:** The use of the word “LEPER” should be avoided, as it is now considered derogatory and dehumanizing.

**Response:** Addressed throughout, as detailed in our responses to Reviewers #1 and #2.

We are grateful to the Editor and reviewers for the opportunity to substantially strengthen this manuscript, and we hope the revised version now meets the journal’s standards for publication.

Sincerely,

The Authors
