# Supplementary material for: Understanding influences on health-seeking behaviours among persons cured of leprosy in the Godokpe community, Volta Region of Ghana: A qualitative descriptive study
Source: PLoS Negl Trop Dis. 2026 Sep 30;20(9):e0014753. doi: 10.1371/journal.pntd.0014753 (PMC13626315; doi:10.1371/journal.pntd.0014753)
Supplement: Supplementary file 5 [file pntd.0014753.s006.docx]

# Response to Reviewers

**Manuscript: Understanding influences on health-seeking behaviours among persons cured of leprosy in the Godokpe community, Volta Region of Ghana: A qualitative descriptive study**

**Reviewers’ comments:**

**Methods**
Reviewer #2: ”Facilitators” added to aims but absent from findings. The third aim now includes “facilitators”, yet the Results report only barriers. If improved institutional treatment is intended as a facilitator, it must be named and discussed as such. Otherwise the aim overpromises.

Response: We thank the reviewer for identifying this inconsistency. On reflection, we have reverted the study’s third aim to its original focus on challenges influencing health-seeking behaviour, rather than retrofit the manuscript’s findings and discussion to a broader claim the study was not designed to substantively support. The corresponding wording in the Abstract has been reverted accordingly. We note, separately, that the study’s inductive analytic approach was not restricted to challenge-oriented codes, and the Social Treatment and Stigma theme in Results does capture a documented shift toward improved institutional treatment of PCLs over time; however, we agree this finding is best presented as part of the study’s broader thematic findings rather than folded into an aim specifically framed around facilitators.

A new theme (Social Treatment and Stigma) appears without explanation. If these data were collected in the original study but unreported in the first manuscript, or were extracted post hoc, a brief statement of transparency is needed. This matters for reader trust.

Response: We thank the reviewer for raising this and are glad to clarify directly. This theme was not derived from any new data collection; the original interview guide already included dedicated questions on participants’ treatment by family, society, and health facility staff, and the underlying transcript material has existed since the original data collection for this study. This material was not identified as a distinct theme in our original analysis. In preparing this revision, we revisited the coded transcripts using the same reflexive thematic analysis procedure described in our Methods section, and the Social Treatment and Stigma theme was developed inductively from this existing dataset. We appreciate the reviewer’s attention to this point and hope this explanation is sufficient; we did not consider a statement to this effect necessary within the manuscript itself, as it pertains to the iterative process of revision rather than to the study’s data collection or design.

The reflexivity statement remains thin for a study of this nature. Researcher gender, linguistic and cultural position relative to Ewe-speaking participants, and orientation toward the supernatural claims that pervade the findings are all unexamined. The limitations section candidly admits that “positionality details such as researcher gender… were not systematically documented”, but gender and language background are factual and recoverable and should be added. Concrete bracketing practices should also be described rather than merely asserted.

Response: We thank the reviewer for this important prompt. We have expanded the Reflexivity section to specify the research assistants’ gender (one male, one female), their status as native Ewe speakers, and their cultural positioning relative to the supernatural explanatory frameworks featured in participants’ accounts. We have also replaced the general assertion of bracketing with a description of the concrete practices used: post-interview debriefing discussions, written memos capturing initial reactions prior to formal coding, and team coding-review meetings in which individual assumptions were explicitly raised and discussed.

Reviewer #3: The objectives are well articulated and the study design appropriate. Population and sampling is appropriate as well. The authors have improved on the description of data elements but may be advised to work on reducing the word count in this section - can consider avoiding quoting the questions verbatim in addition to the descriptions. No outstanding concerns about ethical or regulatory requirements being met.

Response: We thank the reviewer for this suggestion. The Data Collection Instrument and Procedure section has been trimmed by removing the verbatim example questions, while retaining the four-domain structure and the distinction between current and retrospective health-seeking material.

**Results**
Reviewer #2: The “severity hierarchy” interpretation appears imposed rather than demonstrated. The claim that participants navigate health sources by perceived severity is analytically appealing but the quotations do not consistently support it: one participant self-medicates because of denial of care, another uses church exclusively. The framing should either be grounded in explicit participant statements or presented more cautiously as the authors’ interpretive synthesis.

Response: We agree, and on closer review of the underlying transcripts found that a single severity-based hierarchy across all care sources was not consistently supported by participants’ own accounts, as the reviewer notes. We have revised this passage to identify the distinct and sometimes co-occurring logics actually evident in participants’ statements, rather than imposing a single explanatory frame across accounts that do not uniformly support it.

“Cured lepers” appears in the Table 1 heading and body text. These three residual instances should be replaced with person-first language, consistent with the rest of the manuscript.

Response: We thank the reviewer for catching these residual instances, which were missed in our earlier terminology revision. All three have been corrected: the Results subheading now reads “Socio-demographic Characteristics of Persons Cured of Leprosy,” the Table 1 caption now reads “Table 1: Socio-demographic characteristics of persons cured of leprosy (PCLs),” and the opening sentence of the accompanying paragraph now reads “Table 1 presents background characteristics of the persons cured of leprosy (PCLs) who participated in the study.” We have additionally reviewed this paragraph in full and corrected several other residual inconsistencies identified during this check (ethnicity phrasing, occupation phrasing, and verb tense).

Quotation alterations raise concerns about data integrity. Several quotations in the revised manuscript differ substantively from the original version. Participant 2’s quote now includes the claim “The disease is not infectious”, which was absent before. Participant 1’s account of her grandfather diagnosing bewitchment was removed. In their response to the reviewers, the authors state that quotations required “correction for accuracy”. This is a significant admission, yet the corrections were made silently, without acknowledgement. Furthermore, accuracy and length are not mutually exclusive; correcting errors does not preclude providing the fuller narrative extracts originally requested. The authors should either flag these corrections transparently in the manuscript or, if the changes reflect post hoc reinterpretation rather than genuine transcription errors, acknowledge that instead.

Response: We thank the reviewer for pressing on this, and apologize that our previous response letter characterized these changes too briefly to be reassuring. In preparing this revision, we conducted a full audit of every quotation in the Results section against the original interview transcripts. This audit found that several quotations in the original submission had silently combined participants’ answers to different interview questions into what was presented as a single continuous quote, in some cases also omitting or altering clauses from what participants actually said. Participants 1 and 2, specifically identified by the reviewer, are two instances of this pattern: in both cases, we removed material that had been spliced in from participants’ answers to a different question, and restored the complete, accurate answer to the question the quote was actually illustrating. The same audit also identified and corrected sex-attribution errors for three participants, an age inconsistency for one participant, an invented clause not present in the source transcript for one participant, and two quotations that had been substantially expanded beyond what participants actually said. We have corrected all of these against the source transcripts. These were genuine transcription/compilation errors from the original manuscript preparation, not post hoc reinterpretation of participants’ meaning; in every case, the correction was to align the manuscript with what the transcript actually records. We agree that accuracy and length are not mutually exclusive, and confirm that, having completed this audit, we retained the fullest verbatim material genuinely available for each quotation; where participants’ actual answers were brief, we did not lengthen them beyond what was said.

Reviewer #3: The results are well presented and clear.

Reviewer #4: in the table replace no idea with unknown Table 2: formating, make sure the lines are even line 369: Replace this ‘Participant 3’s’ in the quote line 488 add (...)

**Conclusions**
Reviewer #2: (No Response)

Reviewer #3: The discussion section has been tightened and generally flows into the conclusions, which are supported by the data.

Public health relevant is discussed. Although concerns about drawing general conclusions from such a small study in a limited geographic area remain, it is worth noting the novelty of its focus on the post-cure population and the addition of two findings not clearly established in literature - i.e. the varying trajectories of institutional and interpersonal stigma, and practical implementation of welfare/health entitlements for this sub-set of persons within the community.

Response: We thank the reviewer for this observation and are glad these two contributions came through clearly. We agree that the single-site design limits generalisability, and this is stated explicitly in Strengths and Limitations; we hope the findings nonetheless offer a useful, transferable starting point for similar post-cure PCL settings elsewhere.

Reviewer #4: Are there any other studies in Ghana as well that you can use in the discussion? So far there are a lot of studies from very different places.

Response: We thank the reviewer for this suggestion and conducted an additional search specifically for Ghana-based literature. We found that Ghana-based literature on leprosy specifically is limited, and is already represented in this manuscript through the studies most directly relevant to our population and focus: Sottie & Darkey (2019), on Godokpe itself; Ahiabu & Tabong (2024), on contact tracing and community perceptions in Ho; and Bergman, Britton, & Kneck (2018), on nurses’ perspectives on leprosy stigma in Ghana. Beyond these, the Ghana-based leprosy literature we identified consists predominantly of quantitative cross-sectional studies (for example, health-related quality of life surveys among cured leprosy patients), which do not share this study’s qualitative, health-seeking-behaviour focus.

We also identified Ghana-based qualitative studies of health-seeking behaviour in comparable demographic populations (e.g., older, low-income adults) that were not leprosy-specific. Having reflected on this alongside Reviewer #3’s earlier comment cautioning against weak linkage to non-comparable populations, we have chosen not to incorporate these into the Discussion, as doing so would risk reintroducing the same population-mismatch concern in reverse: geographic proximity alone does not make a study a good comparator if its population and focus differ substantially from ours. We have therefore retained the current Discussion’s reliance on leprosy-specific and PCL-specific studies, which we consider the more defensible basis for comparison, even where this means drawing more heavily on international literature than Ghana-specific literature.

**Editorial and Data Presentation Modifications?**

Use this section for editorial suggestions as well as relatively minor modifications of existing data that would enhance clarity. If the only modifications needed are minor and/or editorial, you may wish to recommend “Minor Revision” or “Accept”.

Reviewer #2: (No Response)

Reviewer #3: - Take the name “Cured Leper’s Village” out of title; suggestion: “Godokpe settlement”

Response: We thank the reviewer for this suggestion. We have removed “(the Cured Lepers’ Village)” and “Ho Municipality” from the title, and adopted “the Godokpe settlement, Volta Region of Ghana” in their place.

- One word “factors” will suffice for “facilitators and challenges” on lines 26, 29, 144-145 as well as in the abstract

Response: We thank the reviewer for this suggestion. In response to a related comment from Reviewer #2 regarding the same wording, we had already reverted “facilitators and challenges” to “challenges” alone throughout the manuscript and Abstract, rather than compress it to “factors,” as we judged that the study’s design and findings remain most accurately described as challenges-focused. We hope this resolves the reviewer’s underlying concern about concise, non-overpromising phrasing, even though the specific wording differs from the reviewer’s suggestion.

- Lines 107-108 (Consider reviewing structure of sentence: Suggestion: “...Ghana recorded 278 new cases (8.075 per 1,000,000 population), with a total of 373 cases (prevalence of 10.834 per 1,000,000 population) still remaining on treatment registers as at the end of the year.)

Response: We thank the reviewer for this suggested restructuring, which we have adopted, as it clarifies that the 373 figure represents cases remaining on treatment registers rather than new cases. We have applied the same clarifying structure to the following sentence on grade-2 disabilities, child cases, and female cases, for consistency.

- Concerning reference [8] for statement running from line 110 to 111 - The cited paper is not the original source of this information. It is also noted that this information is not appropriately cited in the source paper. The authors are advised to check this against more recent information.

Response: We thank the reviewer for catching this citation error. We have traced the statement to its correct primary source and updated the citation accordingly.

- Concerning the description of Godokpe on lines 117-118, can we take out the derogatory term altogether? (this repeats itself severally (e.g. line 157, 193, 302-303, 708), it should be possible to avoid using CLV in this publication, given the inclusion of a derogatory disused term within the name.

Response: We thank the reviewer for this important point and agree fully. We have removed “Cured Lepers’ Village” and the abbreviation “CLV” throughout the manuscript, including all five instances identified by the reviewer, using “Godokpe” as the sole place name, with “the community” or “the settlement” used for variation where appropriate. We note one related but distinct term that necessarily remains: “Cured Lepers Foundation” is the actual, formally registered legal name of the third-party non-governmental organisation that facilitated community-level access for this study, as discussed in Ethics Considerations, and we are not in a position to substitute an alternative name for this organisation, as doing so would misidentify the actual entity involved. We have, however, minimised repetition of this full name by referring to it as “the Foundation” after its first mention within each relevant section.

- line 136: instead of “now free of infectivity”, consider “rendered non-infectious after start of MDT” (persons affected by leprosy become non-infectious soon after initiating treatment, way before completing MDT course and being declared cured).

Response: We thank the reviewer for this clinically important correction and have adopted the suggested phrasing, replacing “now free of infectivity” with “rendered non-infectious after starting MDT,” to accurately reflect that non-infectiousness occurs early in treatment rather than only upon completion and cure.

- Line 322-323 - the statement on widows being the majority can be changed to reflect the picture more accurately - Half of the participants were widowed - 9 widows and 1 widower.

Response: Corrected. The socio-demographic summary now states that half of the participants were widowed (nine widows, one widower), accurately reflecting the underlying data rather than characterising this as a majority.

- Line 551-554 - The article cited (reference [10]) is a preprint and has not been peer-reviewed. It reports new medical research that has yet to be evaluated and so should not be used to guide clinical practice.

Response: We thank the reviewer for flagging this. The study has since been peer-reviewed and published; we have updated reference [10] to cite the published version in place of the earlier medRxiv preprint.

Reviewer #4: some changes, I suggest:

- In the abstract, line 23: the sentence that starts with ‘In Ghana’ should be restructured as it is a bit confusing.

Response: Revised. The sentence now separates the two distinct ideas it was previously conflating: that leprosy has been eliminated as a public health problem in Ghana while new cases continue to be detected, and that PCLs separately continue to face long-term stigma, disability, and barriers to healthcare.

- In the abstract use less brackets for listing this.

Response: We have revised the Abstract throughout to convert parenthetical lists into prose, retaining brackets only for the two necessary abbreviation definitions (PCLs, SDG).

- don’t repeat explanations for abbreviation, for example grade-disabilities

Response: We thank the reviewer for this observation and have reviewed the manuscript for abbreviations defined more than once. Grade-2 disabilities (G2D) is now spelled out only at first mention, with “G2D” used thereafter throughout the manuscript, including in the Abstract, Introduction, Results, and Discussion.

- You don’t need to mention Supplementary File_1 in brackets (line 155)

 Response: Removed. The bracketed reference has been deleted from this location; the manuscript retains an explicit pointer to the relevant supplementary file only where it is first substantively described (Data Collection Instrument and Procedure, and Supporting Information legends), consistent with the reviewer’s suggestion to avoid redundant referencing.

**Summary and General Comments**

Use this section to provide overall comments, discuss strengths/weaknesses of the study, novelty, significance, general execution and scholarship. You may also include additional comments for the author, including concerns about dual publication, research ethics, or publication ethics. If requesting major revision, please articulate the new experiments that are needed.

Reviewer #2: (No Response)

Reviewer #3: A much-improved manuscript following the first round of reviews, which has addressed majority of concerns with the discussion section. While presenting some novel findings, concerns remain about the limited geographic scope and small study size for generalizability.

Reviewer #4: - use less explanations in brackets

- don’t mention Supplementary File_1 for example in the text

- in general make sure you have 4 clear themes. mention them in the beginnning, and make yure you keep the wording the same. especially the headings
