# Supplementary material for: Understanding influences on health-seeking behaviours among persons cured of leprosy in the Godokpe community, Volta Region of Ghana: A qualitative descriptive study
Source: PLoS Negl Trop Dis. 2026 Sep 30;20(9):e0014753. doi: 10.1371/journal.pntd.0014753 (PMC13626315; doi:10.1371/journal.pntd.0014753)
Supplement: Peer review history

# Peer review: Decision Letter 0

**DOI:** 10.1371/journal.pntd.0014753.r001

**Authors:** Mathieu Picardeau, Anil Fastenau

2 Jul 2026

PNTD-D-26-01040

Understanding influences on health-seeking behaviours among persons cured of leprosy at the Ho Leprosarium in the Volta Region of Ghana: A phenomenological qualitative study

PLOS Neglected Tropical Diseases

Dear Dr. Delali,

Thank you for submitting your manuscript to PLOS Neglected Tropical Diseases. After careful consideration, we feel that it has merit but does not fully meet PLOS Neglected Tropical Diseases's publication criteria as it currently stands. Therefore, we invite you to submit a revised version of the manuscript that addresses the points raised during the review process.

Please submit your revised manuscript within by Aug 31 2026 11:59PM. If you will need more time than this to complete your revisions, please reply to this message or contact the journal office at plosntds@plos.org. When you're ready to submit your revision, log on to https://www.editorialmanager.com/pntd/ and select the 'Submissions Needing Revision' folder to locate your manuscript file.

Please include the following items when submitting your revised manuscript:

* A letter that responds to each point raised by the editor and reviewer(s). You should upload this letter as a separate file labeled 'Response to Reviewers'. This file does not need to include responses to any formatting updates and technical items listed in the 'Journal Requirements' section below.

* A marked-up copy of your manuscript that highlights changes made to the original version. You should upload this as a separate file labeled 'Revised Manuscript with Track Changes'.

* An unmarked version of your revised paper without tracked changes. You should upload this as a separate file labeled 'Manuscript'.

If you would like to make changes to your financial disclosure, competing interests statement, or data availability statement, please make these updates within the submission form at the time of resubmission. Guidelines for resubmitting your figure files are available below the reviewer comments at the end of this letter.

As the corresponding author, your ORCID iD is verified in the submission system and will appear in the published article. PLOS supports the use of ORCID, and we encourage all coauthors to register for an ORCID iD and use it as well. Please encourage your coauthors to verify their ORCID iD within the submission system before final acceptance, as unverified ORCID iDs will not appear in the published article. Only the individual author can complete the verification step; PLOS staff cannot verify ORCID iDs on behalf of authors.

We look forward to receiving your revised manuscript.

Kind regards,

Anil Fastenau, M.D., M.Sc.

Guest Editor

PLOS Neglected Tropical Diseases

Mathieu Picardeau

Section Editor

PLOS Neglected Tropical Diseases

Shaden Kamhawi

co-Editor-in-Chief

PLOS Neglected Tropical Diseases

orcid.org/0000-0003-4304-636XX

Paul Brindley

co-Editor-in-Chief

PLOS Neglected Tropical Diseases

orcid.org/0000-0003-1765-0002

**Additional Editor Comments (if provided):**

**Journal Requirements:**

If the reviewer comments include a recommendation to cite specific previously published works, please review and evaluate these publications to determine whether they are relevant and should be cited. There is no requirement to cite these works unless the editor has indicated otherwise.

1) We have noticed that you have uploaded Supporting Information files, but you have not included a list of legends. Please add a full list of legends for your Supporting Information files after the references list.

2) In the online submission form, you indicated that Researchers requesting access to the data may be required to provide a research proposal, evidence of institutional ethical approval, a signed data use agreement, and justification demonstrating that the intended use aligns with the ethical conditions under which the original data were collected. The corresponding author, Dickson Kweku Delali (dicksonattu6@gmail.com), serves as the data custodian and can provide further information regarding the data access process.

All PLOS journals now require all data underlying the findings described in their manuscript to be freely available to other researchers, either

1. In a public repository

2. Within the manuscript itself

3. Uploaded as supplementary information.

This policy applies to all data except where public deposition would breach compliance with the protocol approved by your research ethics board. If your data cannot be made publicly available for ethical or legal reasons (e.g., public availability would compromise patient privacy), please explain your reasons by return email and your exemption request will be escalated to the editor for approval. Your exemption request will be handled independently and will not hold up the peer review process, but will need to be resolved should your manuscript be accepted for publication. One of the Editorial team will then be in touch if there are any issues.

3) We have amended your Competing Interest statement to comply with journal style. We kindly ask that you double check the statement and let us know if anything is incorrect.

**Reviewers' Comments:**

Reviewer's Responses to Questions

**Key Review Criteria Required for Acceptance?**

As you describe the new analyses required for acceptance, please consider the following:

**Methods**

-Are the objectives of the study clearly articulated with a clear testable hypothesis stated?

-Is the study design appropriate to address the stated objectives?

-Is the population clearly described and appropriate for the hypothesis being tested?

-Is the sample size sufficient to ensure adequate power to address the hypothesis being tested?

-Were correct statistical analysis used to support conclusions?

-Are there concerns about ethical or regulatory requirements being met?

Reviewer #1: (No Response)

Reviewer #2: The study addresses a genuinely important and under-researched population. The choice of the Ho setting builds directly on prior local research and has strong contextual relevance. Sampling and data management are appropriate, and the attention to COREQ guidelines and trustworthiness demonstrates a genuine commitment to research quality. The phenomenological framing is a promising choice, though there appears to be some mismatch between the claimed design and the analysis as presented. Naming the specific tradition guiding it (e.g., transcendental, hermeneutic, or existential phenomenology) would help readers better understand the analytical lens applied. Alternatively, the authors might consider reframing the study as a qualitative descriptive design using thematic analysis. The objectives as currently stated read as descriptive aims. A phenomenological study might benefit from centring a more specific lived experience question, for example, what it means for cured individuals to navigate health decisions in a context of stigma and segregation. The interview duration appears relatively brief for a phenomenological study. This is not inherently problematic, particularly given the age and potential fatigue of participants, but the authors may wish to acknowledge and briefly justify this duration as a point of transparency. Several participants have unknown ages. Providing estimated ranges (e.g., "approximately 70-80 years") with appropriate caveats would be a helpful addition. Including WHO Disability Grades in the participant table, if/where available, could also offer useful context, as disability severity may shape participants’ experiences of stigma, mobility, and healthcare access in ways that enrich the analysis. Finally, authors should include a reflexivity statement for researchers involved in data collection to describe their positionality, disciplinary backgrounds, and relationship to the community, as these may shape data collection and interpretation.

Reviewer #3: The authors have stated a general aim for the study supported by 3 specific objectives, the last of which suggests they believe that challenges may be affecting health-seeking negatively - this does not however qualify a clear testable hypothesis.

While the qualitative design is appropriate to address the stated objectives, the earlier point on hypothesis and framing of the 3rd specific objectives suggest some bias. How did the authors ensure that positive factors influencing health-seeking were not ignored? It will be helpful to have more detailed description of the elements of their data collection instrument under the relevant section under methodology, as well as include the specific ethics-approved IDI question guide as an attachment with their response.

Sample size (to saturation) and analysis are appropriate for a qualitative study of this nature.

Questions arise from having to obtain approval from the "Cured Leper Foundation." Is this a formal registered entity and is there a legal mandate granting them oversight of free citizens cured of leprosy? If this foundation is related to the philanthropists involved in constructing the home, as stated by lepers, did ownership rights of this public space accrue to them? And will it not then be more appropriate to have a public authority responsible for rights of such a vulnerable population be approached for some approval.

**********

**Results**

-Does the analysis presented match the analysis plan?

-Are the results clearly and completely presented?

-Are the figures (Tables, Images) of sufficient quality for clarity?

Reviewer #1: (No Response)

Reviewer #2: The thematic organisation is clear and logical. Direct quotations ground findings in participant voices. The range of health-seeking sources identified (biomedical, herbal, self-medication, spiritual) paints a useful picture of pluralistic health behaviour. The challenges relating to mobility, poverty, and drug unavailability are vividly illustrated, and the tension between food and healthcare expenses is poignantly captured. The analysis remains descriptive rather than interpretive. Subthemes are presented as lists of what participants said, without exploring underlying meanings, decision-making processes, or patterns across cases. Thematic analysis should move beyond cataloguing responses to interpreting what those responses reveal. A striking analytical opportunity is missed with Participant 3, who uses technical biomedical language (Mycobacterium leprae, airborne disease, sensory neurons) entirely absent from all other accounts. This outlier offers potential for interpretation. Does it reflect differential education, provider contact, or social position? The quotations included offer useful illustrations of participants' views, and the analysis could be strengthened further by incorporating slightly longer extracts that give readers more insight into the narratives and lived experiences behind these responses. The Health-Seeking Sources findings describe where participants go for care but not how they navigate between systems or what meanings these choices hold.

Reviewer #3: It is good to see the authors present summary socio-demographics and thematic findings data in comprehensive table; however, in its current form, the former (Table 1) doesn't have information on educational background, although the discussion points to most of the study population having minimal formal education. Additionally, the repeated use of "thus" in lines 261-263 suggests link to a pre-existing condition satisfied and confuses clear meaning.

**********

**Conclusions**

-Are the conclusions supported by the data presented?

-Are the limitations of analysis clearly described?

-Do the authors discuss how these data can be helpful to advance our understanding of the topic under study?

-Is public health relevance addressed?

Reviewer #1: (No Response)

Reviewer #2: The conclusions are clearly structured, and the recommendations are specific and actionable. Linking findings to SDGs 1, 3, and 10 appropriately frames public health relevance. The emphasis on post-cure support as distinct from active-case management is a valuable contribution. The strengths and limitations section acknowledges several important constraints including single-site design, recall bias, and translation challenges. The claim that the study "breaks new ground by centring phenomenological voices" might overstate the contribution given the methodological concerns outlined above. The limitations could discuss the brevity of interviews. The policy recommendations are sensible but largely reiterate established positions in the leprosy literature. The authors could strengthen the discussion by engaging more deeply with the structural dimensions of the findings. For instance, one participant describes being denied free medications despite an apparent policy of free care, which raises questions about rights and accountability that go unexamined. Future research directions are appropriate, particularly the call for longitudinal work and inclusion of provider and family perspectives.

Reviewer #3: At the high-level, the conclusions are generally support by the data presented. The authors have addressed both public health relevance and how their findings can advance our understanding of this topic.

Nevertheless, the discussion section should be further tightened, avoiding drawing weak linkage such as comparing knowledge/perceptions of healthcare workers who are not normally expected to seek healthcare (the focus of this study) to persons with a lived experience of disease who will normally continue to seek care, and desist from introducing author inferences and opinions which can raise questions on doubts. 3 examples are given below (not by any means, exhaustive, though):

- Lines 467-470: Does Gopalakrishnan et al assess KAP amongst persons with a lived experience of the disease or urban adults generally, and should it be likened to this specific study population of PCLs. Secondly, look at the structure of the sentence. It appears like 2 ideas have been combined without punctuation.

- Lines 475-477: Contrasting the knowledge of medical trainees with interns, which is more likely the impact of medical education (didatic, interact, and/or involving patients), rather than the kind of patient-healthcare provide interactions referred to by the authors. And how do the authors know hospital interactions are/were likely rare; this is not given as a key finding, and the study is not appropriately designed to determine this.

- Line 490-494: The reader may struggle to find why the finding of one woman (out of 20 participants) preferring the church entirely is being compared to findings from a study where initial traditional healers gave way to biomedical care only if ineffective, and how the suggested reason of timing is valid. Even with the explanation of the reason (lines 492-494, the sentence is rather long - are the reader struggles to understand the point being made.

The authors are advised to overhaul the discussion section

**********

**Editorial and Data Presentation Modifications?**

Use this section for editorial suggestions as well as relatively minor modifications of existing data that would enhance clarity. If the only modifications needed are minor and/or editorial, you may wish to recommend “Minor Revision” or “Accept”.

Reviewer #1: (No Response)

Reviewer #2: The manuscript is generally well written. The term "lepers" (line 186) must be replaced with person-first language (PCLs), consistent with terminology used elsewhere in the manuscript and with current standards in the field. Similarly, the repeated use of "Cured Lepers’ Village" throughout the paper could be reconsidered; adopting the local name "Godokpe" after first mention would avoid inadvertently reinforcing the very stigma the study seeks to address. Authors may wish to consider whether consistently attributing quotations to numbered participants alongside age and sex could risk deductive disclosure in a small, identifiable community. A brief statement on how this was managed would be reassuring. A few instances of phrasing could be softened for sensitivity, for example, describing participants' situation as a "predicament" (line 425) carries a tone that could be more neutral.

Reviewer #3: The use of the word "LEPER" should be avoided, as it is now considered derogatory and dehumanizing.

**********

**Summary and General Comments**

Use this section to provide overall comments, discuss strengths/weaknesses of the study, novelty, significance, general execution and scholarship. You may also include additional comments for the author, including concerns about dual publication, research ethics, or publication ethics. If requesting major revision, please articulate the new experiments that are needed.

Reviewer #1: (No Response)

Reviewer #2: This manuscript addresses an important and under-researched topic: the post-cure experiences of persons affected by leprosy in a segregated community in Ghana. The focus on health-seeking behaviour among this marginalised population is commendable and aligns well with the WHO Global Leprosy Strategy 2021-2030 emphasis on stigma reduction, disability management, and social inclusion. However, the manuscript has several significant methodological, analytical, and presentational weaknesses that require substantial revision before it can be considered for publication. The core concerns fall into three interrelated domains: conceptual clarity and use of terminology, methodological rigour and alignment with the claimed phenomenological design, and analytical depth and presentation of findings. Addressing these issues would allow the study's genuine strengths to come through more effectively. These strengths include its unique setting, its engagement with a hard-to-reach population, and the relevance of its policy recommendations.

Reviewer #3: Generally, a relevant study that addresses key issues which can improve the quality of healthcare and welfare of persons with a lived experience of leprosy (whether with active disease or long after cure). Study is a small one limited in geographic scope which makes findings not readily generalizable, particularly when the discussion section has not adequately situated findings within larger body of evidence - this section should be overhaul to more appropriately compare findings in this specific population with SIMILAR populations (PCLs) from other studies in other geographic regions, or with other cured patient populations (for other disease, or active leprosy vs PCLs).

**********

PLOS authors have the option to publish the peer review history of their article (what does this mean?). If published, this will include your full peer review and any attached files.

If you choose “no”, your identity will remain anonymous but your review may still be made public.

**Do you want your identity to be public for this peer review?**  For information about this choice, including consent withdrawal, please see our Privacy Policy.

Reviewer #1: No

Reviewer #2: No

Reviewer #3: No

[NOTE: If reviewer comments were submitted as an attachment file, they will be attached to this email and accessible via the submission site. Please log into your account, locate the manuscript record, and check for the action link "View Attachments". If this link does not appear, there are no attachment files.]

**Figure resubmission:**

**Reproducibility:**

To enhance the reproducibility of your results, we recommend that authors of applicable studies deposit laboratory protocols in protocols.io, where a protocol can be assigned its own identifier (DOI) such that it can be cited independently in the future. Additionally, PLOS ONE offers an option to publish peer-reviewed clinical study protocols. Read more information on sharing protocols at https://plos.org/protocols?utm_medium=editorial-email&utm_source=authorletters&utm_campaign=protocols

Attachment Submitted filename: Review for PLOS-NTDs.docx

# Author comment: Author response to Decision Letter 1

**DOI:** 10.1371/journal.pntd.0014753.r002

26 Jul 2026

Attachment Submitted filename: Response to Reviewers.docx

# Peer review: Decision Letter 1

**DOI:** 10.1371/journal.pntd.0014753.r003

**Authors:** Stuart Blacksell, Anil Fastenau

2 Sep 2026

Response to ReviewersRevised Manuscript with Track ChangesManuscript'.

Shaden Kamhawi

co-Editor-in-Chief

PLOS Neglected Tropical Diseases

orcid.org/0000-0003-4304-636XX

Paul Brindley

co-Editor-in-Chief

PLOS Neglected Tropical Diseases

orcid.org/0000-0003-1765-0002

**Reviewers' comments:**

**Key Review Criteria Required for Acceptance?**

As you describe the new analyses required for acceptance, please consider the following:

**Methods**

-Are the objectives of the study clearly articulated with a clear testable hypothesis stated?

-Is the study design appropriate to address the stated objectives?

-Is the population clearly described and appropriate for the hypothesis being tested?

-Is the sample size sufficient to ensure adequate power to address the hypothesis being tested?

-Were correct statistical analysis used to support conclusions?

-Are there concerns about ethical or regulatory requirements being met?

Reviewer #2: “Facilitators” added to aims but absent from findings. The third aim now includes “facilitators”, yet the Results report only barriers. If improved institutional treatment is intended as a facilitator, it must be named and discussed as such. Otherwise the aim overpromises.

A new theme (Social Treatment and Stigma) appears without explanation. If these data were collected in the original study but unreported in the first manuscript, or were extracted post hoc, a brief statement of transparency is needed. This matters for reader trust.

The reflexivity statement remains thin for a study of this nature. Researcher gender, linguistic and cultural position relative to Ewe-speaking participants, and orientation toward the supernatural claims that pervade the findings are all unexamined. The limitations section candidly admits that “positionality details such as researcher gender… were not systematically documented”, but gender and language background are factual and recoverable and should be added. Concrete bracketing practices should also be described rather than merely asserted.

Reviewer #3: The objectives are well articulated and the study design appropriate. Population and sampling is appropriate as well. The authors have improved on the description of data elements but may be advised to work on reducing the word count in this section - can consider avoiding quoting the questions verbatim in addition to the descriptions. No outstanding concerns about ethical or regulatory requirements being met.

Reviewer #4: Yes, the method is well described an clear.

Explain long-term cured, is there a definition? A clear time line?

You don't need all the example interview questions

Who was present at the interviews?

Who translated for participants who were not able to speak english?

Who were the t research assistants? Nationality?

**********

**Results**

-Does the analysis presented match the analysis plan?

-Are the results clearly and completely presented?

-Are the figures (Tables, Images) of sufficient quality for clarity?

Reviewer #2: The “severity hierarchy” interpretation appears imposed rather than demonstrated. The claim that participants navigate health sources by perceived severity is analytically appealing but the quotations do not consistently support it: one participant self-medicates because of denial of care, another uses church exclusively. The framing should either be grounded in explicit participant statements or presented more cautiously as the authors' interpretive synthesis.

“Cured lepers” appears in the Table 1 heading and body text. These three residual instances should be replaced with person-first language, consistent with the rest of the manuscript.

Quotation alterations raise concerns about data integrity. Several quotations in the revised manuscript differ substantively from the original version. Participant 2’s quote now includes the claim “The disease is not infectious”, which was absent before. Participant 1’s account of her grandfather diagnosing bewitchment was removed. In their response to the reviewers, the authors state that quotations required “correction for accuracy”. This is a significant admission, yet the corrections were made silently, without acknowledgement. Furthermore, accuracy and length are not mutually exclusive; correcting errors does not preclude providing the fuller narrative extracts originally requested. The authors should either flag these corrections transparently in the manuscript or, if the changes reflect post hoc reinterpretation rather than genuine transcription errors, acknowledge that instead.

Reviewer #3: The results are well presented and clear.

Reviewer #4: in the table replace no idea with unknown

Table 2: formating, make sure the lines are even

line 369: Replace this 'Participant 3’s'

in the quote line 488 add (...)

**********

**Conclusions**

-Are the conclusions supported by the data presented?

-Are the limitations of analysis clearly described?

-Do the authors discuss how these data can be helpful to advance our understanding of the topic under study?

-Is public health relevance addressed?

Reviewer #2: (No Response)

Reviewer #3: The discussion section has been tightened and generally flows into the conclusions, which are supported by the data.

Public health relevant is discussed. Although concerns about drawing general conclusions from such a small study in a limited geographic area remain, it is worth noting the novelty of its focus on the post-cure population and the addition of two findings not clearly established in literature - i.e. the varying trajectories of institutional and interpersonal stigma, and practical implementation of welfare/health entitlements for this sub-set of persons within the community.

Reviewer #4: Are ther any other studies in Ghana as well that you can use in the discussion? So far there are a lot of studies from very different places.

**********

**Editorial and Data Presentation Modifications?**

Use this section for editorial suggestions as well as relatively minor modifications of existing data that would enhance clarity. If the only modifications needed are minor and/or editorial, you may wish to recommend “Minor Revision” or “Accept”.

Reviewer #2: (No Response)

Reviewer #3: - Take the name "Cured Leper's Village" out of title; suggestion: "Godokpe settlement"

- One word "factors" will suffice for "facilitators and challenges" on lines 26, 29, 144-145 as well as in the abstract

- Lines 107-108 (Consider reviewing structure of sentence: Suggestion: "...Ghana recorded 278 new cases (8.075 per 1,000,000 population), with a total of 373 cases (prevalence of 10.834 per 1,000,000 population) still remaining on treatment registers as at the end of the year.)

- Concerning reference [8] for statement running from line 110 to 111 - The cited paper is not the original source of this information. It is also noted that this information is not appropriately cited in the source paper. The authors are advised to check this against more recent information.

- Concerning the description of Godokpe on lines 117-118, can we take out the derogatory term altogether? (this repeats itself severally (e.g. line 157, 193, 302-303, 708), it should be possible to avoid using CLV in this publication, given the inclusion of a derogatory disused term within the name.

- line 136: instead of "now free of infectivity", consider "rendered non-infectious after start of MDT" (persons affected by leprosy become non-infectious soon after initiating treatment, way before completing MDT course and being declared cured).

- Line 322-323 - the statement on widows being the majority can be changed to reflect the picture more accurately - Half of the participants were widowed - 9 widows and 1 widower.

- Line 551-554 - The article cited (reference [10]) is a preprint and has not been peer-reviewed. It reports new medical research that has yet to be evaluated and so should not be used to guide clinical practice.

Reviewer #4: some changes, I suggest:

- In the abstract, line 23: the sentence that starts with 'In Ghana' should be restructured as it is a bit confusing.

- In the abstract use less brackets for listing this.

- don't repeat explanations for abbreviation, for example grade-disabilities

- You don't need to mention Supplementary File_1 in brackets (line 155)

-

**********

**Summary and General Comments**

Use this section to provide overall comments, discuss strengths/weaknesses of the study, novelty, significance, general execution and scholarship. You may also include additional comments for the author, including concerns about dual publication, research ethics, or publication ethics. If requesting major revision, please articulate the new experiments that are needed.

Reviewer #2: (No Response)

Reviewer #3: A much-improved manuscript following the first round of reviews, which has addressed majority of concerns with the discussion section. While presenting some novel findings, concerns remain about the limited geographic scope and small study size for generalizability.

Reviewer #4: - use less explanations in brackets

- don't mention Supplementary File_1 for example in the text

- in general make sure you have 4 clear themes. mention them in the beginnning, and make yure you keep the wording the same. especially the headings

**********

PLOS authors have the option to publish the peer review history of their article (what does this mean?). If published, this will include your full peer review and any attached files.

If you choose “no”, your identity will remain anonymous but your review may still be made public.

**Do you want your identity to be public for this peer review?**  For information about this choice, including consent withdrawal, please see our Privacy Policy.

Reviewer #2: No

Reviewer #3: No

Reviewer #4: No

**Figure resubmission:**

**Reproducibility:** -->To enhance the reproducibility of your results, we recommend that authors of applicable studies deposit laboratory protocols in protocols.io, where a protocol can be assigned its own identifier (DOI) such that it can be cited independently in the future. Additionally, PLOS ONE offers an option to publish peer-reviewed clinical study protocols. Read more information on sharing protocols at https://plos.org/protocols?utm_medium=editorial-email&utm_source=authorletters&utm_campaign=protocols-->?>

# Author comment: Author response to Decision Letter 2

**DOI:** 10.1371/journal.pntd.0014753.r004

13 Sep 2026

Attachment Submitted filename: Response_to_Reviewers_auresp_2.docx

# Editor report: Decision Letter 2

**DOI:** 10.1371/journal.pntd.0014753.r005

**Authors:** Stuart Blacksell, Anil Fastenau

16 Sep 2026

Dear Mr Delali,

We are pleased to inform you that your manuscript 'Understanding influences on health-seeking behaviours among persons cured of leprosy in the Godokpe community, Volta Region of Ghana: A qualitative descriptive study' has been provisionally accepted for publication in PLOS Neglected Tropical Diseases.

Before your manuscript can be formally accepted you will need to complete some formatting changes, which you will receive in a follow up email. A member of our team will be in touch with a set of requests.

Please note that your manuscript will not be scheduled for publication until you have made the required changes, so a swift response is appreciated.

IMPORTANT: The editorial review process is now complete. PLOS will only permit corrections to spelling, formatting or significant scientific errors from this point onwards. Requests for major changes, or any which affect the scientific understanding of your work, will cause delays to the publication date of your manuscript.

Should you, your institution's press office or the journal office choose to press release your paper, you will automatically be opted out of early publication. We ask that you notify us now if you or your institution is planning to press release the article. All press must be co-ordinated with PLOS.

Thank you again for supporting Open Access publishing; we are looking forward to publishing your work in PLOS Neglected Tropical Diseases.

Best regards,

Anil Fastenau, M.D., M.Sc.

Guest Editor

PLOS Neglected Tropical Diseases

Stuart Blacksell

Section Editor

PLOS Neglected Tropical Diseases

Shaden Kamhawi

co-Editor-in-Chief

PLOS Neglected Tropical Diseases

orcid.org/0000-0003-4304-636XX

Paul Brindley

co-Editor-in-Chief

PLOS Neglected Tropical Diseases

orcid.org/0000-0003-1765-0002

***********************************************************

p.p1 {margin: 0.0px 0.0px 0.0px 0.0px; line-height: 16.0px; font: 14.0px Arial; color: #323333; -webkit-text-stroke: #323333}span.s1 {font-kerning: none

# Editor report: Acceptance letter

**DOI:** 10.1371/journal.pntd.0014753.r006

**Authors:** Stuart Blacksell, Anil Fastenau

Dear Mr Delali,

We are delighted to inform you that your manuscript, "Understanding influences on health-seeking behaviours among persons cured of leprosy in the Godokpe community, Volta Region of Ghana: A qualitative descriptive study," has been formally accepted for publication in PLOS Neglected Tropical Diseases.

We have now passed your article onto the PLOS Production Department who will complete the rest of the publication process. All authors will receive a confirmation email upon publication.

The corresponding author will soon be receiving a typeset proof for review, to ensure errors have not been introduced during production. Please review the PDF proof of your manuscript carefully, as this is the last chance to correct any scientific or type-setting errors. Please note that major changes, or those which affect the scientific understanding of the work, will likely cause delays to the publication date of your manuscript. Note: Proofs for Front Matter articles (Editorial, Viewpoint, Symposium, Review, etc...) are generated on a different schedule and may not be made available as quickly.

Soon after your final files are uploaded, the early version of your manuscript will be published online unless you opted out of this process. The date of the early version will be your article's publication date. The final article will be published to the same URL, and all versions of the paper will be accessible to readers.

For Research Articles, you will receive an invoice from PLOS for your publication fee after your manuscript has reached the completed accept phase. If you receive an email requesting payment before acceptance or for any other service, this may be a phishing scheme. Learn how to identify phishing emails and protect your accounts at https://explore.plos.org/phishing.

Thank you again for supporting open-access publishing; we are looking forward to publishing your work in PLOS Neglected Tropical Diseases.

Best regards,

Shaden Kamhawi

co-Editor-in-Chief

PLOS Neglected Tropical Diseases

Paul Brindley

co-Editor-in-Chief

PLOS Neglected Tropical Diseases
